# Resolving whether inhalation of depleted uranium contributed to Gulf War Illness using high-sensitivity mass spectrometry

**DOI:** 10.1038/s41598-021-82535-3

**Published:** 2021-02-18

**Authors:** Randall R. Parrish, Robert W. Haley

**Affiliations:** 1 School of Environment, Geography and Geosciences, University of Portsmouth, Burnaby Building, Portsmouth, PO1 3QL UK; 2 Division of Epidemiology, Department of Internal Medicine, University of Texas Southwestern Medical Center, 5323 Harry Hines Blvd., Dallas, TX 75390 USA

**Keywords:** Environmental sciences, Biomarkers, Diseases, Mass spectrometry

## Abstract

Of the hypothesized causes of Gulf War Illness (GWI), a chronic multi-symptom illness afflicting approximately 25% of military personnel deployed to the 1991 Gulf War, exposure to depleted uranium (DU) munitions has attracted international concern. Past research has not tested the potential association of GWI with inhaled DU nor used isotope mass spectrometry of sufficient sensitivity to rigorously assess prior DU exposure. We applied a standard biokinetic model to predict the urinary concentration and uranium isotopic ratios for a range of inhalation exposures. We then applied sensitive mass spectrometry capable of detecting the predicted urinary DU to 154 individuals of a population-representative sample of U.S. veterans in whom GWI had been determined by standard case definitions and DU inhalation exposures obtained by medical history. We found no difference in the ^238^U/^235^U ratio in veterans meeting the standard case definitions of GWI versus control veterans, no differences by levels of DU inhalation exposure, and no ^236^U associated with DU was detected. These findings show that even the highest likely levels of DU inhalation played no role in the development of GWI, leaving exposure to aerosolized organophosphate compounds (pesticides and sarin nerve agent) as the most likely cause(s) of GWI.

## Introduction

Thirty years have passed since the 1990 invasion of Kuwait by Iraq and the subsequent 1991 Persian Gulf War that liberated Kuwait. During or shortly after the 1991 conflict, an estimated 25%^1^ of the approximately 700,000 deployed U.S. (and additional allied) military personnel developed an unusual chronic multi-symptom illness, referred to as Gulf War Illness (GWI)^2^, manifested by fatigue, fever and night sweats, memory and concentration problems, pathogen-free diarrhoea, sexual dysfunction, chronic body pain and other symptoms compatible with autonomic nervous system dysfunction^3^ and dysfunction of the brain’s cholinergic system^4^. Investigations into the causes have considered potential war theatre exposures including low-level chemical warfare nerve agent(s), pyridostigmine bromide anti-nerve-agent medication, pesticides, multiple immunizations, depleted uranium (DU), and combat stress. The possibility of toxic effects from DU, first raised after the first large scale use of DU munitions in the Gulf War, have stimulated international concern because of potential exposure of civilian populations from the Gulf War and from later conflicts in Kosovo, Bosnia, the Persian Gulf and others. Studies attempting to address this concern have failed to generate a consensus because of limitations in the sensitivity of tests for DU in urine and the lack of any investigation of DU in veterans meeting accepted case definitions of GWI.

DU is uranium depleted isotopically in the more fissile ^235^U isotope which is separated by isotope enrichment methods to produce enriched ^235^U for use in nuclear reactors and nuclear weapons. Instead of remaining as unused nuclear waste, it has been made into dense armour-piercing munitions used in military conflicts in 1991 and 2003 onwards as well as in tank armour. In a hard target impact a DU-containing projectile efficiently penetrates the target’s armour, partially fragments the DU core, and ignites a brief intense fire, combusting and oxidizing DU into aerosolized oxides. Humans may then internalize DU by inhalation of aerosolized DU oxides, oral ingestion of DU oxide particles that settle in the environment, or retention of DU in metallic form in shrapnel fragments in body wounds.

Adverse effects of significant intake are hypothesized to result from heavy metal toxicity and alpha particle radiation from DU mainly in the lungs, kidneys and bone where it is concentrated. Despite numerous studies demonstrating long-term urinary excretion of DU from industrial exposure and prediction of possible adverse effects on the basis of doses of heavy metal and alpha radiation^5–8^, no actual adverse effects in humans have been described, though serious effects may be masked because of major gaps in research^9^. Nevertheless, DU continues to be considered a plausible cause of GWI^10^ and courts in the UK and Italy have attributed illness and death to inhalation exposure to DU^11,12^.

The 1991 Gulf War presents an opportunity to study the health effects of inhaled DU. Approximately 300 tonnes of DU-munitions were fired by tanks, artillery and aircraft mainly at targets in southern Iraq and especially along the Basra Road where Iraqi tanks were destroyed in large numbers by DU munitions. In addition to combat-related exposures, in July 1991 a post-war explosion and fire at an ammunition storage site within U.S. Army Camp Doha, Kuwait, resulted in a series of explosions involving several tonnes of DU munitions, causing DU-related aerosols to be released into the dense smoke plume from the fire to which many personnel were exposed during containment and clean-up^13^.

Natural uranium (NU) is composed of ^238^U (99.27%), ^235^U (0.72%) and ^234^U (0.0054% in minerals, 0.005–0.02% in water sources), with a ^238^U/^235^U ratio of 137.80–137.88^1,14^. In the production of nuclear fuel, enriched uranium (EU) high in ^235^U is extracted from NU leaving the residual DU strongly depleted in ^235^U and thus with a greatly increased ^238^U/^235^U ratio of approximately 500^8,15^. Aside from fission of ^235^U in power generation, nuclear reactions also include neutron capture by ^235^U generating ^236^U, which is absent in NU; when re-enriched during nuclear fuel recycling, the resulting DU acquires this rare isotope. While EU has variable and significant amounts of ^236^U, DU contains ^236^U in low proportions (~ 0.003%)^15^. The large differences in isotope composition among EU, NU and DU can be exploited by mass spectrometry to quantify even small proportions of DU in humans and the environment^16,17^.

NU occurs naturally in food, water and soil. A portion (~ 2%) of ingested or inhaled uranium is absorbed into the bloodstream, concentrated and stored in bone and kidney, and excreted slowly in urine over many years. A large intake of NU, EU or DU can usually be detected by a urine assay for total uranium concentration [U]. Total [U] values above 43 ng/g of creatinine—the 95th percentile of the US population^18,19^—indicate an excess body load of uranium, but highly sensitive mass spectrometry is required to determine whether the excess is due to NU, EU, DU or some combination. Two methods of mass spectrometry have been used to detect DU in human urine samples: lower precision sector-field mass spectrometry (SF-ICP-MS) has been used to differentiate DU from NU in Gulf War veterans at ^238^U/^235^U ratios above 166; whereas, higher precision multi-collector mass spectrometry (MC-ICP-MS) applied to chemically purified U can detect DU at ^238^U/^235^U ratios as low as 140.

A series of studies between 1993 and 2009 was conducted to determine whether an exposure to DU in the 1991 Gulf War resulted in enough absorption to detect in urine years later^5^. At first, small numbers of U.S. soldiers who had been involved in friendly fire explosions and, later, U.S. veterans who wanted to be tested provided urine samples for assay. All of the studies attempted to identify DU by measuring the total [U]. Some, but not all, veterans with X-ray-documented DU fragments retained in their bodies were found to have extremely high total [U] levels, far above the 95th percentile of the U.S. population—the cut point for distinguishing DU—and no veterans with inhalation exposures but no retained shrapnel did. In recent studies^20,21^, however, measurements with low precision SF-ICP-MS identified 3 veterans out of 1,700 with ^238^U/^235^U ratio above the cut point of 166 confirming DU in their urine, but 2 of these 3 had total [U] below the cut point for DU and the third was right at the cut point; whereas, 29 veterans with no evidence of DU by SF-ICP-MS were nevertheless above the cut point by total [U], and other veterans who recalled inhalation exposures had far lower total [U] levels. Thus, while these studies demonstrated that a few veterans with retained shrapnel excrete very high levels of DU in their urine, the method was too imprecise to evaluate excretion from inhalation exposures. More importantly, since none of the studies measured the veterans’ continuing symptoms or applied the standard GWI case definitions, no study has yet addressed the question of whether the symptoms of GWI could be due to DU exposure.

In this paper we extend prior work to measurement of DU excretion in U.S. veterans with or without GWI who reported information on possible DU exposures. We first calculated the urinary DU concentrations expected as a function of time since exposure to plausible DU oxide levels from aerosol inhalation scenarios in the 1991 Gulf War^6,8,22^ based on the Human Respiratory Tract Model (HRTM) relating absorption and excretion^23^. We then evaluated the capability of the following 3 bioassay methods to detect the subtle DU excretion in urine expected from inhalation absorption: (1) screening for excess total [U]; (2) measurement of ^238^U/^235^U ratio with high sensitivity multi-collector mass spectrometry (MC-ICP-MS) on chemically purified U; and finally, bivariate analysis of the ^236^U/^238^U and ^235^U/^238^U isotopic ratios. We applied these methods to a highly studied population-representative sample of both theatre-deployed and non-deployed Gulf War-era U.S. veterans in whom Gulf War Illness (GWI) had been determined by standard case definitions, whose wartime DU exposures had been ascertained by interview, and who provided a 24-h urine sample 18–20 years after the war. We compared these measured values to those predicted to be found 18–20 years after exposure to medically meaningful DU levels in the war. The goal was to provide the best chance of finding DU if potential disease-causing inhalation exposure had occurred and for the first time provide high confidence in a negative finding.

## Results

### Predicted values of DU excretion from historic inhalation exposures

Inhaled fine particulates of DU oxide have the potential to lodge deep in the lung, decompose slowly into solution into the blood stream, and become stored in bone, kidney and other organs to be excreted over time with organ and bone re-working, all while undergoing slow radioactive decay. The rate of dissolution is a key parameter that underpins the notion that DU can be detected in urine many years after exposure.

To test whether DU plays any role in GWI, it is first necessary to predict the concentration of DU expected in a urine sample for a given DU aerosol inhalation exposure as a function of the dose and oxide type of the original DU exposure, the time since exposure, and likely dietary intakes of NU in the run up to urine collection. Without this prediction, it is impossible to know whether a negative test is due to an inconsequential exposure or an insensitive urine assay method—a valid criticism of past research on this problem.

The defining studies by the U.S. Department of Defense^6^, the World Health Organisation^22^ and the British Royal Society^8^, classified battlefield situations of potential DU aerosol exposure into 3 standard exposure levels likely to occur in combat situations: level I-high, level II-medium, and level III-low. Exposure level I includes direct inhalation of an impact aerosol; level II, inhalation of resuspended impact aerosol or oral ingestion within a contaminated vehicle; and level III, inhalation of an aerosol plume at a distance from an impact or fire or resuspension from ground contamination. Doses in milligrams of DU taken into the body in each of these situations were estimated with evidence from other studies, including field studies involving measurements during destruction of vehicles with DU penetrators (Table 1)^6^.

**Table 1:** Current ranges of urinary total [U] and U isotopic ratios that identify DU estimated from different levels of DU inhalation exposure during the 1991 Gulf War, time since exposure, and daily dietary intake of natural uranium running up to urine sample collection.

Parameters specified in the prediction model			Ranges of parameters expected 18 years after specified DU inhalation exposure			Can expected levels of DU be detected by criteria used in prior studies?		
Standard DU exposure level	Estimated DU intake (mg) for the exposure level in 1991 Gulf War^a^	Daily dietary U excretion (*n*g) in run-up to testing	Total [U] (*n*g U/g creatinine)	^238^U/^235^U	^236^U/^238^U × 10^–6^	Method 1: total [U] > 50 ng U/g creatinine^b^	Method 2: ^238^U/^235^U > 166 by SF-ICP-MS^c^	Method 3: ^238^U/^235^U > 140 by MC-ICP-MS^d^
Level I	250	2	14.5–74.5	367–467	25.9–29.2	Rarely	Yes	Yes
		8	20.5–80.5	247–397	18.3–27.0	Rarely	Yes	Yes
Level II	10	2	2.5–4.9	161–241	6.0–17.8	No	Yes	Yes
		8	8.5–10.9	144–171	1.8–8.0	No	No	Yes
Level II	5	2	2.3–3.5	150–198	3.3–12.6	No	No	Yes
		8	8.3–9.5	141–155	0.9 -4.6	No	No	Yes
Level III	2	2	2.1–2.6	143–165	1.4–6.7	No	No	Yes
		8	8.1–8.6	139–145	0.4–2.0	No	No	Yes

^a^Numerical values are derived from calculations of^8,23,24^ as illustrated in Fig. 1, described in more detail in on-line methods.

^b^In the most recent study of DU in Gulf War veterans, Dorsey et al.^20^ screened urine total [U] for DU by this criterion.

^c^Dorsey et al.^20^ identified the presence of DU with the ^238^U/^235^U ratio measured by lower precision sector field–inductively coupled plasma-mass spectrometry (SF-ICP-MS).

^d^The present study identified the presence of DU with the ^238^U/^235^U ratio measured by the high precision multiple collector–inductively coupled plasma-mass spectrometry (MC-ICP-MS).

**Figure 1:**
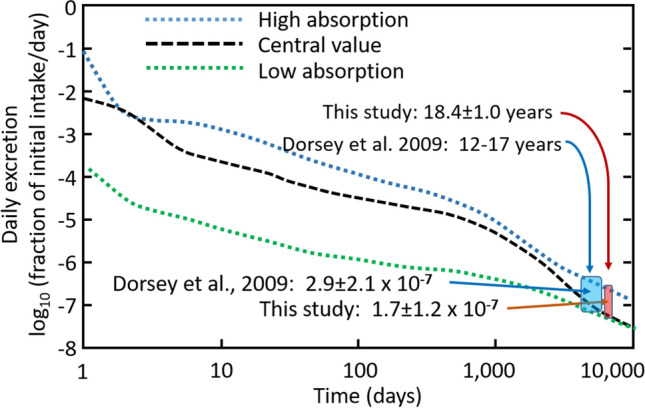
Estimation of current urinary DU excretion from DU oxides absorbed 18 years earlier. From the Human Respiratory Track Model (HRTM) for inhalation of uranium oxides we estimated the daily urinary excretion of DU, expressed as a fraction of the original inhaled dose (log scale), and plotted as a function of days since inhalation (log scale). In the sample of veterans studied, the duration between potential inhalation (in the first half of 1991 during the conflict) and the urine collection (November 2008–June 2010) was 6720 ± 365 days or 18.4 ± 1.0 years, shown as the width of the small red box. The analogous region from Dorsey et al.^20^ discussed in the text is shown in the larger blue box. Three curves showing fractional uranium excretion predicted by the combined dissolution-storage-excretion function of the HRTM for uranium oxide particulates are shown; these represent “Low” and “High” absorption curves during dissolution and metabolism of compounds of uranium oxide with contrasting chemical form and solubilities. The figure is modified from^8,24^ using the HRTM model with aerosol and oxide physicochemical characteristics summarized^8^ and taking into account the uncertainty in the HRTM model parameters^23^.

We used the modelling of^8,22,24^ to estimate the urinary excretion of uranium on the basis of an inhalation dose. We considered intake doses representing each of the Royal Society’s exposure levels at 18 years after exposure and at 2 likely dietary intake levels of NU in the run up to urine collection to predict current excretion rates of DU oxides (UO_2_, U_3_O_8_, etc.) in a range of excretion levels determined by the level of uncertainty in the ICRP models (Fig. 1). The model output^8,24^ allowed calculation of the current expected ranges of the following 3 parameters most used for detecting past DU exposure from urinary assays: total [U], the ^238^U/^235^U isotopic ratio, and the ^236^U/^238^U ratio, given in Table 1.

### Comparison of the ability of three methods to distinguish DU from NU in urine

#### Total [U]

Applying the criterion of finding > 50 ng U/g creatinine of total [U] in urine will detect only veterans excreting the very highest total [U] in those with Level I inhalation exposures in the Gulf War, but would be unable to distinguish any with Level II or III exposures from NU excretion (Table 1).

#### ^238^U/^235^U measured by SF-ICP-MS

The relatively low precision SF-ICP-MS method would be able to differentiate DU from NU in all veterans with Level I exposures in the Gulf War and most in the upper half with Level II exposures but only if they are consuming a diet low in NU (average 2 ng NU per day) so that DU is ≥ 25% of the total U excreted. It would thus be unable to differentiate most with Level II and all with Level III exposures from NU excretion (Table 1).

#### ^238^U/^235^U measured by MC-ICP-MS after rigorous chemical purification of U (this study)

The high precision MC-ICP-MS method combined with chemical separation and purification of U from urine, allows the detection of excreted DU at a rate of > 0.068 ng/day, our methodological DU detection limit. Our prediction model indicates that it would detect an initial inhalation exposure of as little as 0.40 mg of DU from the 1991 Gulf War. Thus, it would be able to identify DU in all veterans with Gulf War exposures far less than the lowest Level III exposure (2 mg DU inhaled during the Gulf War) even if consuming a diet relatively high in NU before urine collection (Table 1). It follows then that only mass spectrometry with high precision MC-ICP-MS is capable of detecting DU from inhalation exposure in the Gulf War or confirming its absence in most Gulf War veterans.

### Cohort of GWI cases and controls and battlefield deployment

We measured the uranium isotope composition in urine samples from a nested case–control sample of Gulf War veterans selected in a 3-stage stratified random sample of the 1991 U.S. military population studied in the U.S. Military Health Survey (USMHS). The methods of sample selection at the 3 stages have been published^3,25^. The first stage involved a computer-assisted telephone interview (CATI) survey (N = 8020) which included questions covering battlefield situations likely to involve inhalation of various levels of DU, from which we assigned the standard DU exposure levels. The second stage involved blood collection from all veterans whose symptoms met the 3 widely used case definitions of GWI (cases) and an approximately 15% random sample of those not meeting it (controls), including both deployed and not deployed to the Kuwaiti Theatre of Operations. The third stage constituted a smaller representative sample selected from the larger stage 2 sample, and included 106 who met the 3 standard case definitions of GWI^2,26,27^ in which cases comprised of 31 with syndrome variant 1 (“impaired cognition”); 42 with syndrome variant 2 (“confusion-ataxia”); 33 with syndrome variant 3 (“central pain”)^2^; and 47 control veterans comprised of 26 deployed to the war theatre and 21 non-deployed not meeting the case definitions. Between November 2008 and June 2010, the 154 veterans in the stage 3 sample were studied extensively in a 7-day clinical research protocol in which each travelled to Dallas, Texas (USA) to be hospitalized in the University of Texas Southwestern Medical Center’s Clinical and Translational Research Center. In addition to diverse neuropsychological, autonomic and neuroimaging studies, a 24-h urine sample was collected in urine containers prewashed with nitric acid to remove any trace uranium; creatinine was measured on an aliquot shortly after collection.

### Effectiveness of total [U] of urine to rule out past DU exposure

**Figure 2:**
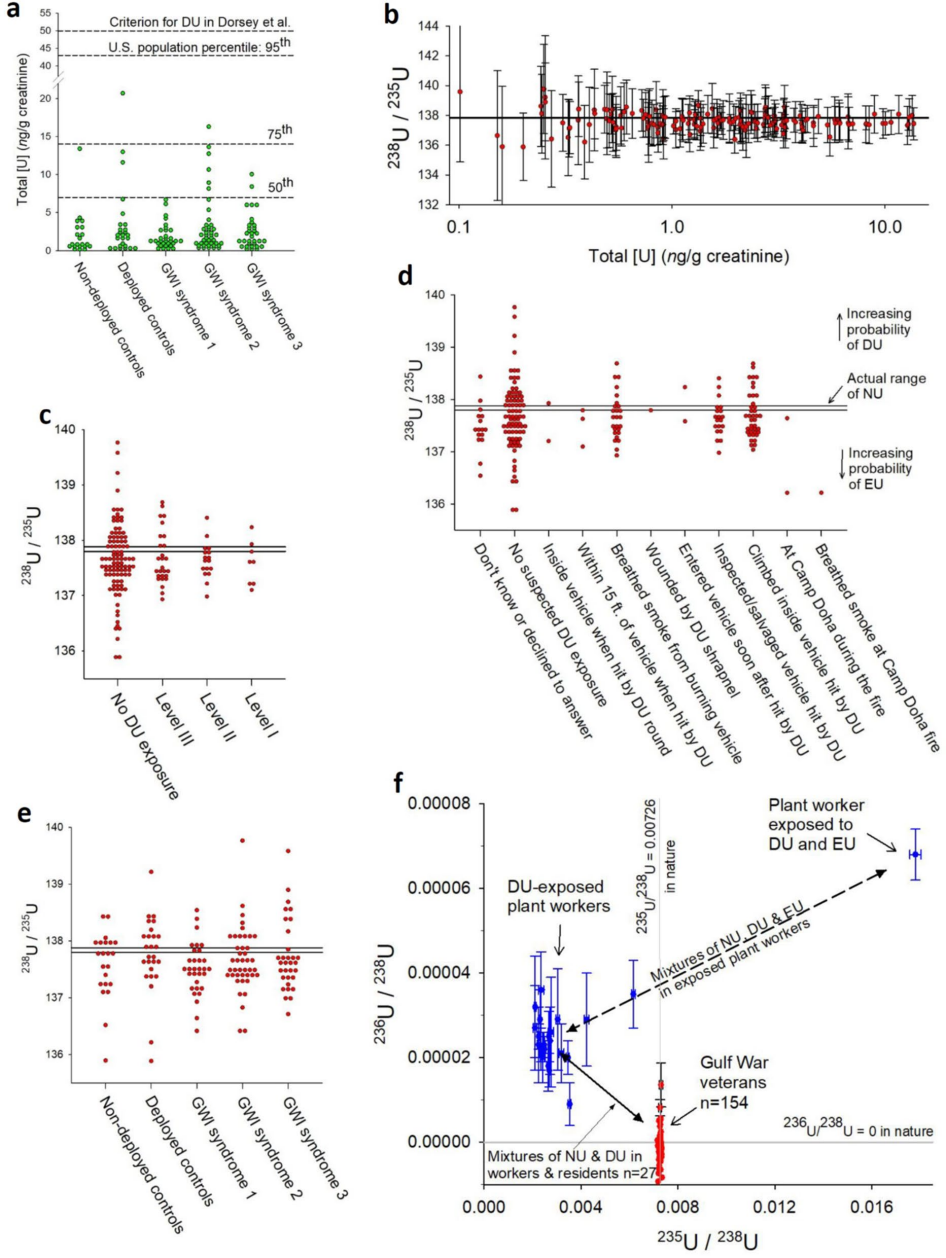
Exposure levels, incidents, and GWI clinical classification. (**a**) Total [U] (*ng/*g of creatinine) by veterans’ GWI clinical classification; horizontal lines indicate the 50th, 75th and 95th percentiles of the distribution of urine U concentrations in the US population^18,19^ and the lower limit for detecting DU of^20^. (**b**) Veterans’ ^238^U/^235^U ratios with individual measurement uncertainty intervals by total [U], demonstrating how the uncertainty intervals and variance increase at lower total [U]. All certainty intervals overlap natural U (horizontal lines). (**c–e**) Distributions of veterans’ ^238^U/^235^U ratios by standard classification of DU exposure levels^8^, (**c**) by specific DU exposure situations (**d**), and by GWI clinical classification (**e**). The horizontal reference lines indicate the range of the ^238^U/^235^U ratio of natural U (137.80–137.88), above which values suggest mixtures of natural U and DU and below which, mixtures of natural and enriched U; individual measurement uncertainties of ~ 1% are omitted for clarity. (**f**) Bivariate scatterplot of ^236^U/^238^U ratio by ^235^U/^238^U ratio for the sample of Gulf War veterans (red symbols), overlaid by those of workers in a DU plant in the town of Colonie, New York, known to have had substantial occupational inhalation DU exposures^7,28^. The horizontal reference line at ^236^U/^238^U = 0 emphasizes that natural U contains no ^236^U. The ratios from subjects with various exposure levels of DU would fall along the solid line; whereas, subjects with mixed exposure to DU and enriched U would fall in the upper right where one such DU worker is shown. Six of the Gulf War veterans had elevated ^236^U/^238^U ratios with certainty intervals that do not overlap zero, but their ^235^U/^238^U ratios are not reduced, which is incompatible with DU and EU; this is caused by an isobaric interference on mass 236 and is explained in the online methods and the text. The standard DU exposure levels range from level III (lowest) to level I (highest)^8^ The GWI clinical classification includes syndrome 1 (impaired cognition), syndrome 2 (confusion-ataxia) and syndrome 3 (central pain)^2^.

The first method we used to detect evidence of past DU exposure was to screen the 154 GWI cases and control subjects’ urine for an increase in total [U] excretion (Table 2). The distribution of total [U] was similar to that of the U.S. population^19^, and no values exceeded its 95^th^ percentile (Fig. 2a). The geometric mean of total [U] of the cases meeting the 3 case definitions of GWI was 1.78 ng U/g creatinine, statistically indistinguishable from the combined deployed and non-deployed controls (geometric mean 1.57 ng U/g creatinine, t test *P* = 0.18). These values of total [U] are consistent with lack of DU contamination but do not exclude the possibility of the small amounts of DU expected from our prediction model with Level II and III inhalation exposures (Table 1). Although continuing dissolution of DU shrapnel in metallic form retained in the body usually increases total [U] beyond the population’s 95% percentile, as concluded by^20^, our findings further confirm that screening of total [U] is not useful for detecting the far smaller intake doses and the time-limited exposure situations involved in inhalation exposures to DU aerosols (Table 1).

**Table 2:** Urinary excretion rate of total U by Gulf War veterans’ clinical group.

Clinical group	Sample size	Total [U]				Total [U] adjusted for creatinine	
		Mean	95% CI	5th and 95th percentile	Range	Median	Mean
Non-deployed controls	21	2.37	− 1.04/+ 1.86	0.24–11.2	0.2–30.2	1.25	1.24
Deployed controls	27	3.80	− 1.60/+ 2.78	0.42–50.2	0.32–96.4	2.16	1.87
GWI syndrome 1	31	2.53	− 0.78/+ 1.12	0.58–10.7	0.22–13.3	1.38	1.41
GWI syndrome 2	42	4.00	− 1.25/+ 1.81	0.77–21.7	0.70–28.8	2.05	2.15
GWI syndrome 3	33	3.33	− 1.05/+ 1.54	0.65–12.0	0.44–15.1	1.95	1.74
All controls	49	2.98	− 0.98/+ 1.45	0.36–25.9	0.17–96.4	1.86	1.57
All GWI	106	3.30	− 0.57/+ 0.69	0.69–16.2	0.13–23.9	1.84	1.78
All samples	154	3.24	− 0.56/+ 0.68	0.57–17.6	0.17–96.4	1.84	1.71

### Use of high precision ^238^U/^235^U to determine significant inhalation exposure

The second method we used to detect past DU exposure was analysis of the ^238^U/^235^U isotopic ratio measured by the high precision MC-ICP-MS method (Table 3). The uncertainty in the ^238^U/^235^U ratio, measured by its 95% confidence interval, increases (precision decreases) as the urinary total [U] decreases (Fig. 2b), but it was less than ± 1% for 95% of the samples (Fig. 2b). For urine samples with total [U] above 1 ng/g creatinine and a ± 1% uncertainty, values of the ^238^U/^235^U ratio above 139–140 including their lower uncertainty bound are considered likely to represent the presence of DU (Fig. 2b). This threshold of 139–140 for confirmation of DU is more robust and > 10 times more sensitive than the threshold of 166 applied by Dorsey et al. using the lower precision SF-ICP-MS (Table 1)^20^.

**Table 3:** Urinary uranium isotope ratios by Gulf War veterans’ clinical group.

Clinical group	Sample size	^238^U/^235^U			^236^U/^238^U	
		Median	Mean^a^	95% CI^a^	Mean^a,c^	95% CI^a^
Undeployed controls	21	137.79	137.62	0.26	< LOD	n/a
Deployed controls	27	137.84	137.75	0.25	< LOD	n/a
GWI syndrome 1	31	137.50	137.51	0.16	< LOD	n/a
GWI syndrome 2	42	137.62	137.68	0.16	< LOD	n/a
GWI syndrome 3	33	137.62	137.71	0.19	< LOD	n/a
All controls	49	137.79	137.70	0.18	< LOD	n/a
All GWI	106	137.55	137.64	0.10	< LOD	n/a
All samples	154	137.63	137.66	0.09	< LOD	n/a
In house urine no IRMM184^d^	4	137.70	137.68	0.27	< LOD	n/a
2 ppb IRMM184 + 233U^e^	125	137.70	137.71	0.05	1.11E−07	1.3E−08
IRMM184 certified^f^			137.70	0.04	1.25E−07	5.3E−10
Natural uranium			137.82	~ 0.06	< 10^–8^	n/a
DU in munitions			~ 500		~ 0.000030	

*DU* depleted uranium, *IRMM* European Commission's Institute for Reference Materials and Measurements, *LOD* limit of detection, *n/a* not applicable, *U* uranium.

^a^Geometric means with 95% CI.

^b^Since ^238^U/^235^U uncertainties are not significantly asymmetrical and are reported as a single value.

^c^The ^236^U/^238^U ratios were overwhelmingly below the 0.0000015 limit of detection after all corrections and uncertainty propagations were made.

^d^The 4 analyses of in-house urine that had no additional IRMM184 added.

^e^IRMM184+ ^233^U values reported are corrected for very minor contributions of other isotopes in the ^233^U that was added.

^f^IRMM certified value from https://crm.jrc.ec.europa.eu/p/40454/40475/By-application-field/Nuclear/IRMM-184-URANIUM-238-NATURAL-ISOTOPIC-NITRATE-SOLUTION/IRMM-184.

#### Exposure situations

When predictions of DU excretion are applied to groups of veterans with different standard levels of DU inhalation exposures, the ^238^U/^235^U ratios are below 139 for veterans with all 3 inhalation exposure levels (Fig. 2c; Kruskal–Wallis test *P* = 0.74). The 3 veterans with outlying values nearest the threshold between 139 and 140 all had very low values of total [U] and thus very wide uncertainty intervals that generously overlap the ^238^U/^235^U of NU (~ 137.8). When the battlefield exposure situations from which the standard exposure levels were generated were broken out, the distributions of the ^238^U/^235^U ratios showed no pattern suggesting any departure from NU outside of methodological uncertainty (Fig. 2d).

#### GWI symptoms

Likewise, veterans who continue to have potentially disabling symptoms of GWI had distributions of the ^238^U/^235^U ratio that did not differ from the deployed and non-deployed control veterans (Fig. 2e), and none had values that differed from NU. Moreover, the distribution of the ^238^U/^235^U ratios in the 3 GWI syndrome variant groups combined was similar to those of the deployed and non-deployed control groups (Kruskal–Wallis test *P* = 0.16).

### Bivariate analysis of the ^236^U/^238^U and ^235^U/^238^U as a further test of DU exposure

As a further test for the possibility of DU in these veterans, we studied their location on the bivariate distribution of the veterans’ ratios of both ^236^U/^238^U and ^235^U/^238^U isotopic ratios. This is an important procedure because, since ^236^U is found only in EU and DU but not in NU, it provides a further direct method of distinguishing DU from NU and from ascertaining whether EU may also have been involved. Since ^236^U constitutes only ~ 0.003% of DU used in munitions, it can only be measured by high precision MC-ICP-MS and thus has not previously been measured in research on GWI.

We applied this approach to our representative sample of Gulf War veterans in the context of prior studies^7,15,28^ of groups of U.S. civilians exposed to DU (and EU) aerosol pollution in New York State from a uranium fabrication plant in the 1960s and 1970s^29^ and in part studied with MC-ICP-MS of urine samples using the same methods as in our study^7^. These studies demonstrated that significant doses of DU aerosols inhaled by factory workers and residents living nearby can be detected in urine ≥ 25 years after the plant was closed^7,28^, broadly consistent with predictions of the HRTM model.

In the bivariate plot in Fig. 2f, an individual excreting pure NU would be located at the point where ^235^U/^238^U = 0.00726 and ^236^U/^238^U = 0, reflecting the usual amount of ^235^U and the absence of ^236^U in natural U. A theoretical individual excreting pure DU would be located at a point defined by ^235^U/^238^U ≈ 0.002 and ^236^U/^238^U ≈ 0.00003, but most DU-exposed workers excrete a combination of DU and NU, which displaces them downward and to the right; whereas, EU added to the mix would displace them upward and to the right. Using published analyses of workers from the plant, mixtures of NU and DU had lower ^235^U and higher ^236^U, locating them along the solid line. Mixtures of NU and EU would have ^235^U/^238^U above that of NU (^235^U/^238^U > 0.00726), indicating substantially increased ^235^U, and variably increased ^236^U. Mixtures of NU, DU and EU would appear along the dashed line in the diagram.

Published studies of the DU plant workers and local town residents with proven non-military DU aerosol exposure^7,28^ are shown to illustrate where subjects with proven inhalation exposure to DU typically fall. All but 6 of the Gulf War veterans in our study are located in a narrow zone located exactly at ^235^U/^238^U = 0.00726, the value of natural U, and with ^236^U/^238^U ratios within the uncertainty zone around ^236^U/^238^U = 0 (Fig. 2f). The 6 exceptions have slightly elevated ^236^U/^238^U ratios and values of the ^235^U/^238^U incompatible with both DU and EU, indicating an artefact of measurement from organic molecule interference in these samples (see explanation in Supplemental materials, mass spectrometry).

### Veteran with a Level I battlefield exposure and DU shrapnel wound

One Gulf War veteran in our study was standing on an Abrams tank when it was hit in a “friendly fire” accident by a DU round which destroyed the tank, threw the individual several meters, peppered him with a mix of sand, pebbles and shrapnel, tattooing his skin and embedding 2 pea-sized pieces of DU shrapnel under his skin. He breathed the hot gases from the explosion for several minutes. Upon return to his base in the U.S. 4 months later, the shrapnel was removed. During his wartime deployment to the war theatre, however, he was also exposed to low-level sarin nerve agent, took pyridostigmine tablets, and has moderately low PON1 type Q isoenzyme level, which are typical risk factors for GWI^30–32^. The veteran had symptoms satisfying the case definitions of GWI, subclassified as variant syndrome 1 (“cognitive impairment”), but his urine showed a total [U] of 1.35 ng/g creatinine, a ^238^U/^235^U ratio of 137.8, and a ^236^U/^238^U ratio below detection limit (< 0.000001)—all typical of natural U with no DU. If residual DU were present it was being excreted at a rate of < 0.068 ng/day, our limit of detection, and our prediction model indicates that he could have absorbed no more than 0.40 mg of DU from the Gulf War from both inhalation and DU shrapnel.

## Discussion

Our findings, using high precision mass spectrometry MC-ICP-MS on chemically purified U that detects even the lowest level of DU exposure capable of causing illness, demonstrate that a sample of veterans drawn from a large population-representative sample of Gulf War veterans, meeting the case definitions of GWI and reporting a range of inhalation exposures to DU in friendly fire accidents, did not absorb even the smallest amount of DU capable of producing chronic adverse effects on health. The past studies of DU in Gulf War veterans have shown clear evidence of DU absorption into the body only in individuals with chunks of DU in metallic form as shrapnel retained in tissues from friendly fire wounds. The methods of detecting DU used in those studies—total [U] and limited use of U isotope ratios measured by the less precise SF-ICP-MS mass spectroscopy method—however, had insufficient precision to differentiate the lower levels of DU absorption likely with inhalation exposures from dietary NU absorption. Moreover, no past study has tested the association of urinary DU excretion measurements with GWI defined by the standard case definitions. Consequently, past studies did not address the question more widely concerning to Gulf War veterans and others of whether inhalation of DU in the war caused, or contributed to, the GWI. Our study, however, found no DU excretion in either veterans meeting the case definitions of GWI or control veterans not meeting them. Given the high precision of our methods, our results not only show an absence of evidence for an association but evidence for the absence of that association.

The element of our study design that gives meaning to a negative finding is that we first developed estimates of the amount of DU that would still be excreted in urine 18 years after exposure to amounts of DU found through simulation studies to result from various situations where soldiers inhaled DU oxides from explosions of DU munitions. We then tested urine from a representative sample of ill and well Gulf War veterans with the high precision mass spectrometry method, not used in prior testing of large samples of veterans, that is capable of detecting the levels of DU excretion predicted by the model. We based the predictions on the best estimates of the bodily absorption of DU that results from various exposure events and the modelling on the most widely accepted approach of the International Commission on Radiological Protection’s (ICRP) Human Respiratory Tract Model^8,23,24^. This study design provided the best chance of finding evidence of DU exposure if it exists but also provides a high degree of confidence in a negative finding as well.

A limitation of our study is that we included only Gulf War-era veterans who were potentially exposed to inhalation of DU from infrequent, very brief, though often intense, friendly fire accidents and sporadic inhalation of dust potentially contaminated with DU particulates over a 4–5 month period. While study of these Gulf War veterans addresses the question of whether short-term and potentially intense inhalation exposures produce enough systemic absorption to cause chronic illness, it may not adequately address the wider implications of decades of continuous exposure to DU-containing dust faced by the populations in war zones where DU munitions have been deployed. The design of our study applying high precision mass spectroscopy to urine samples could readily be adapted to study residents of former war zones to identify ongoing DU excretion as a quantitative biomarker of true DU absorption. Any effects of this exposure on health could then be addressed with clinical studies comparing sensitive measures of adverse health effects in persons with DU-positive and DU-negative test results.

For now, however, Gulf War veterans need no longer be concerned that a connection between their chronic illnesses and DU exposure in the Gulf War has been inadequately addressed by insensitive methods of measuring DU excretion and studies not relating DU measurements directly to GWI. If DU had been an important cause of GWI, our study, applying the most precise measure of DU to a representative sample of Gulf War veterans meeting the standard case definitions, was sensitive enough to have found it. From our negative results, we conclude that inhalation of DU during the Gulf War was not an important contributor to the GWI. Now scientific attention must be focused even more intensely on the remaining likely causes, particularly the widespread exposure to cholinesterase-inhibiting toxicants including low-level sarin nerve gas known to have been widely dispersed during destruction of chemical weapons stores by Allied bombing of Iraqi chemical weapons storage depots^32,33^, as well as pesticides and pyridostigmine, for which considerable evidence exists^31^.

## Methods

### Subjects

The 154 Gulf War-era U.S. military veterans who participated in this study were selected by a 3-stage statistical sampling plan as a representative sample of those who served in the U.S. armed forces during the 1991 Persian Gulf War, the target population. The first stage involved a computer-assisted telephone interview (CATI) survey of a stratified random sample of the target population, known as the U.S. Military Health Survey (USMHS). The sample was selected from the computer personnel file of the Gulf War-era military population between August 2, 1990 and July 1, 1991, obtained from the Defense Manpower Data Center (DMDC, Seaside, CA), stratified by the following design parameters: a flag indicating deployment to the Kuwaiti Theatre of Operations (KTO), age (< 49 years, ≥ 49 years), sex, race/ethnicity (non-Hispanic white, other), military rank during the war (officer, enlisted), military component (active duty, Reserve/Guard), military occupation (air flight crew, aircraft maintenance, army special operations, other), location in KTO on 20 January 1991 (deployed only), and 3 special study samples (twin pairs, member of 24th Reserve Naval Mobile Construction Battalion, and parent of a child with Goldenhar complex birth defect). Of the full USMHS sample (n = 8020), 6497 were deployed to the KTO, and 1523 were non-deployed. With 74.9% of the selected veterans located and contacted and 80.2% of these agreeing to participate, the overall response rate was 60.1%. The methods, extensive pilot testing and initial findings of the USMHS were described in detail elsewhere^25^.

The standardized interview included questions on specific in-theatre scenarios previously defined by the U.S. Army’s DU Capstone project for calculating the 4 levels of DU exposure recommended for epidemiologic studies by^6,8^. It also contained all questions required to define the 3 most often used case definitions of GWI: the Factor^2^, CDC^26^, and Modified Kansas^27^ definitions. The Factor case definition was developed with principal components analysis of symptom scales to identify groups of veterans with similar patterns related to deployment and was extensively validated^25,34^. All deployed personnel were present for the 5-week air war and the 5-day ground war in January and early February 1991.

The second stage involved selection of all veterans who met any of the 3 case definitions and an approximately 15% random sample of all who did not. The resulting sample included 2103 veterans, from whom samples of peripheral blood serum, plasma, DNA and RNA were collected by trained phlebotomists in or near subjects’ homes, shipped overnight to the UT Southwestern laboratory, and archived for later studies.

The third stage selected a subset (n = 154) of those who participated in the second stage and were randomly selected as a representative sample of Gulf War-era veterans with and without GWI. These were hospitalized between late 2008 and June 2010 in the UT Southwestern Medical Center’s Clinical and Translational Research Center for a 7-day research protocol involving 25 clinical neurological, neuroimaging, EEG and genetic studies of GWI. This protocol included the collection of a 24-h timed urine collection supervised and timed by professional research nurses in the hospital’s clinical research center. The urine samples were collected in urine collection bottles that had been pre-washed with an HCL solution to remove any trace of uranium. Urine samples thus post-date potential DU exposure by approximately 18 years. At the completion of collection, the volume of each 24 h urine sample was measured and recorded, and an aliquot was sent to Quest Laboratories for creatinine determination. The urine samples were then stored at 4 °C. In late 2017, a 500 ml aliquot was taken from each well shaken urine sample, and sent to the University of Portsmouth in the U.K. for uranium isotope analysis with generic sample numbers for blind analysis. All participants gave written informed consent according to a protocol approved by the institutional review board of the University of Texas Southwestern Medical Center and methods followed relevant guidelines and regulations.

### Calculation of inhalation exposure and excretion of inhaled uranium

The predictions of urinary DU concentrations from DU absorbed 18 years earlier, shown in Fig. 1, are based on information in the annexes to the comprehensive study by the Royal Society report on DU [annexes C, G, H of 8; 22,24]. These sources describe the absorption of inhaled material from the respiratory tract and how this can be modelled in terms of accumulation in kidney and bone and excretion in urine over time, dependent upon the type and solubility of uranium oxide particles. These are based upon the Human Respiratory Tract Model (HRTM) of the International Commission on Radiological Protection^23^ and^8^, the latter providing available information on absorption characteristics (i.e. lung solubility) of particulate DU from DU penetrator impact and combustion in fires. Because relatively insoluble UO_2_ and U_3_O_8_ are the predominant oxides from DU^6,35^, they have the slowest dissolution rate constants, approximately 0.0012–0.00035 and 0.0015–0.00049 for U_3_O_8_ and UO_2_, respectively.

The modelling^8,24^ using the Human Respiratory Tract Model^23^ is shown in Fig. 1 for inhalation of U oxides of various types and illustrates daily excretion as a fraction of the original intake as a function of time. The red box of Fig. 1 uses 18.4 ± 1.0 years as the duration between inhalation and urine collection, and the fractional excretion of inhaled DU is 1.7 ± 1.2 × 10^–7^. An analogous blue box is shown for the Dorsey et al. study^20^. Using a range of initial inhalation doses of DU from 250 down to 2 mg, we calculated the DU excretion during the time window of urine collection and added two contrasting ranges of daily dietary excretion of natural U of 2 and 8 ng/day of U. For these calculations the ^238^U/^235^U and ^236^U/^238^U for DU were 500 and 30 × 10^–6^, and for natural U 137.82 and 0, respectively. From these excretion rates, we calculated (Supplemental Material, indicative calculation) the fractional amount of DU in urine, and the corresponding isotope ratios of ^238^U/^235^U and ^236^U/^238^U; this allows assessment of measurement capabilities and limits of detection for DU for our method and that of all previous urinary U isotope measurements of US Gulf War veterans^20^, which used a less sensitive SF-ICP-MS methodology.

The predicted isotope ratios ^238^U/^235^U and ^236^U/^238^U were calculated from Eqs. (1) and (2), using end member DU of ^238^U/^235^U = 500 and ^236^U/^238^U = 30 × 10^–6^, (2) NU ^238^U/^235^U = 137.82 and ^236^U/^238^U = 0, and the fraction of excretion that is DU (*f*_*DU*_).

1 $$^{{{238}}} {\text{U}}/^{{{235}}} {\text{U}}_{{{\text{mixture}}}} = f_{DU} /\left( {^{{{238}}} {\text{U}}/^{{{235}}} {\text{U}}_{{{\text{DU}}}} } \right) + ({1 } - f_{DU} )/\left( {^{{{238}}} {\text{U}}/^{{{235}}} {\text{U}}_{{{\text{NU}}}} } \right)$$

2 $$^{{{236}}} {\text{U}}/^{{{238}}} {\text{U}}_{{{\text{mixture}}}} = f_{DU} \times \left( {^{{{236}}} {\text{U }}/^{{{238}}} {\text{U}}_{{{\text{DU}}}} } \right)$$

The sensitivity threshold for positive detection of DU using ^238^U/^235^U measurements was 139–140 for our method (described below) and 166 according to the methods used by^20^. Because we could not detect DU if its proportion was < 2%, corresponding to a ^238^U/^235^U of 140, we calculate that based on the mean rate of excretion of 3.4 ng/day, the maximum amount of DU excreted would be < 0.068 ng/day. Using the HRTM-based calculation, this leads to a maximum inhalation dose 0.4^+1.0/−0.2^ mg during the Gulf War.

### Chemical and isotope measurement procedures

Samples were measured blind at the University of Portsmouth in that they had generic sample numbers without any information as to subject criteria in terms of deployed, not deployed, Gulf War Illness positive or negative—information which was confidentially retained at the University of Texas Southwestern Medical Center. Upon receipt of samples at the University of Portsmouth, weights of samples were measured and stored for subsequent analysis in a restricted access class 100 laboratory. The general procedures used to measure the concentration and isotope composition of uranium from urine are adapted from prior publications^7,36^ and followed methods in accordance with regulations. Particular variations to published methods are described below and in the Supplemental Material. The procedure involved a full chemical separation and purification of uranium extracted by calcium phosphate co-precipitation from 150 ml of urine using pre-cleaned reagents (see Supplemental Material, reagents) in a class-100 clean laboratory and multiple collector inductively coupled plasma mass spectrometry (MC-ICP-MS). The study used a Nu Instruments MC-ICP-MS with multiple faraday and ion counting detectors, extensive analysis of a 2 ng/g solution of certified IRMM184 (Institute of Reference Materials and Measurement, Geel, Belgium) uranium solution almost identical with natural uranium to which a high isotopic purity ^233^U tracer was added (^238^U/^233^U ~ 100), repeated analysis of an in-house urine to which IRMM184 was partly added, and routine measurement of mass 237 important for making time-dependent corrections to ^236^U/^238^U related to mass abundance sensitivity (down-mass tailing corrections) arising from pressure variations within the mass spectrometer and trace amounts of organic molecular ion interference at masses 236.8 and 235.8 (Supplemental Material, mass spectrometry). The method resulted in a LOD on the ^236^U/^238^U of 0.000015 or 11 counts/sec on the 236 mass. The uranium introduced during the chemical procedure was 11–38 pg averaging 8–12 pg during each batch. Once all of these corrections were made, the analyses of the IRMM184 reference solution yielded ^234^U/^238^U, ^235^U/^238^U and ^236^U/^238^U values of 5.344 × 10–5 ± 2.0 × 10–6, 0.0072614 ± 0.0000025 and 1.1 × 10^–7^ ± 0.1 × 10^–7^, respectively, in agreement with the certified values. The mean ^235^U/^238^U measurement uncertainty and its standard deviation for 154 sample and 125 IRMM184 measurements was 0.66 ± 0.46% and 0.44 ± 0.19%, respectively, with individual analyses available in Supplemental Material Tables S3 and S4.

## Supplementary Information

Supplementary Information 1.

Supplementary Information 2.

Supplementary Information 3.
