# Supplementary material for: Resolving whether inhalation of depleted uranium contributed to Gulf War Illness using high-sensitivity mass spectrometry
Source: Sci Rep. 2021 Feb 18;11:3218. doi: 10.1038/s41598-021-82535-3 (PMC7893152; doi:10.1038/s41598-021-82535-3)
Supplement: Supplementary file 1 — Supplementary Information 1. [file 41598_2021_82535_MOESM1_ESM.docx]

**Resolving whether inhalation of depleted uranium contributed to Gulf War Illness using high-sensitivity mass spectrometry**

**Supplemental Material**

Randall R. Parrish^1*^ and Robert W. Haley^2^

^1^School of Environment, Geography and Geosciences, University of Portsmouth, Burnaby Building, Portsmouth, PO1 3QL, UK

^2^Division of Epidemiology, Department of Internal Medicine, University of Texas Southwestern Medical Center, Dallas, TX 75390, USA

^*^Address correspondence to R. Parrish, School of Environment, Geography and Geosciences, University of Portsmouth, Burnaby Building, Portsmouth, PO1 3QL, UK. Email: [randall.parrish@port.ac.uk](mailto:randall.parrish@port.ac.uk)

**Introduction**

This document describes the method for calculation of (1) predicted U concentration and isotope ratios on the basis of solubility of particles, (2) dietary U intake and isotope ratios of NU and DU, (3) the materials, reagents and methods used in the chemical separation and purification of uranium, and (4) mass spectrometry procedures including instrument type, ion collector configuration and operating conditions for mass spectrometry, and the order of corrections to raw data to obtain final isotope measurements. These materials include tables of operating conditions (**Table S1**), ion counting collector array (**Table S2**), and final data tables for all samples (**Table S3**) and IRMM84 reference solutions (**Table S4**). The supplementary figures illustrate the tailing and organic molecular ion interference on mass 237&236 pertinent to the measurement of the sample and IRMM84 ^236^U/^238^U ratios (**Figures S1-S5**).

**Indicative calculation of inhalation exposure using the Human Respiratory Tract Model (HRTM) of the International Commission on Radiological Protection (ICRP)**

The annexes to the comprehensive studies by the Royal Society on DU [1] and World Health Organisation [2], and [3] describe the absorption of inhaled material from the respiratory tract and how this can be modelled in terms of accumulation in kidney and bone and excretion in urine over time, dependent upon the type and solubility of uranium oxide particles. These are based upon the Human Respiratory Tract Model (HRTM) of the International Commission on Radiological Protection [4]. Annex A of the [1] contains HRTM models used to assess intakes of uranium; Annex C of [1] and [3] model excretion of inhaled uranium oxide, and annexes G and H of [1] contain summaries of available information on absorption characteristics (i.e. lung solubility) of particulate DU from DU penetrator impact and combustion in fires, respectively. Because relatively insoluble UO_2_ and U_3_O_8_ are the predominant oxides from DU combustion [5,6], they have the slowest dissolution rate constants, approximately 0.0012-0.00035 and 0.0015-0.00049 for U_3_O_8_ and UO_2_, respectively. The modelling [1,3] is shown in **figure 1** (main paper) for inhalation of U oxides of various types and illustrates daily excretion arising from the inhaled particle dissolution expressed as a fraction of the original intake as a function of time, which is termed *f_DU_*.

The duration between a brief exposure in the first half of 1991 during the Gulf War and the time window of urine collection in the study, 18.4±1.0 years, is shown by the width of the red box in **figure 1** (main paper), with a excretion fraction value of 1.7±1.2 x 10^-7^ and its uncertainty given by the vertical range of values of the box that overlap all of the uranium oxide solubility curves. An analogous blue box is shown in **figure 1** (main paper) for the Dorsey et al. [7] study. Using a range of initial inhalation doses of DU from 250 to 2 mg, two contrasting ranges of daily dietary intake of natural U of 2 and 8 ng/day, and values of ^238^U/^235^U and ^236^U /^238^U for DU and NU of 500 and 30 x 10^-6^, and 137.82 and 0, respectively, we calculated the DU excretion and its U isotope ratios 18.4±1.0 years later (Table 1 main paper). The equations for calculation of isotope ratios are:

^238^U/^235^U_mixture_ = [*f_DU_* / {^238^U/^235^U_DU_} + (1- *f_DU_* / {^238^U/^235^U_NU_})]^-1^ (1)

^236^U/^238^U_mixture_ = *f_DU_ ** {^236^U /^238^U_DU_} (2)

**Plastic- and glassware and laboratory environment.** Plasticware consisted of Eppendorf pipettors and pipette tips, PFA Teflon or LDPE bottles for storage, HDPE 250ml bottles, 10ml tapered PE columns from Dowex, Quartz glass beakers for wet-ashing and evaporation, PFA Savillex or quartz glass beakers for evaporation, and FEP-enclosed magnet stirring bars. All plastic- and glassware was pre-cleaned with nitric acid and rinsed with purified water. All chemical procedures were done in a class 100 clean lab containing a fume extraction cupboard and a separate HEPA filtered class 100 dedicated clean air hood.

**Reagents.** Water was from a Milli-Q system with 18.2Ω resistivity. Acids comprised HNO_3_ and HCl and were sPA Romil reagents, diluted as appropriate. Ammonia and H_2_O_2_ were also sPA Romil reagents. Ion exchange resins used were 50-100µm TRU, UTEVA and PFR resins from Eichrom. Co-precipitation and other reagents comprised Ca(NO_3_)_2_, ammonium phosphate monohydrate (NH_4_H_2_PO_4_), and Al(NO_3_)_3_. These latter reagents were not sufficiently clean (i.e. free of uranium) to permit off the shelf use and had to be cleaned by dissolution in 3N HNO3 and either mixed in a slurry with TRU +/- UTEVA resin(s) or repeatedly loaded and rinsed through columns containing TRU and UTEVA to remove U which is retained at this normality of nitric acid. Ca(NO_3_)_2_ and Al(NO_3_)_3_ were easy to clean but with the high phosphate content of ammonium phosphate, the retention of U in UTEVA and TRU resins is degraded, so up to 3 additional steps were necessary and even with this level of cleaning, this reagent contributed a majority of the U introduced in the procedure.

**Tracer.** A high purity ^233^U tracer from the IRMM (Institute of Reference Materials and Measurements, Geel, Belgium) was used. Its isotope purity is 99.6% ^233^U; other ratios as measured were: ^235^U/^233^U, 4.81 x 10^-5^; ^236^U/^233^U, 6.0 x 10^-7^; ^238^U/^233^U, 0.005630. This solution was prepared to 0.84ng/ml and dissolved in 1N HNO3. Using a pipettor, 0.045ml, or about 38 pg of ^233^U, was nominally dispensed to samples.

**In-house urine sample.** A several litre in-house urine sample was prepared, aliquoted, and analysed for reproducibility. Three of every four of these reference samples had ~ 0.4, 0.8, and 1.2 ng of IRMM184 uranium added at the stage in the procedure when addition of acid, tracer and co-precipitating reagents was done.

**IRMM84 solution.** A ~2*n*g/g solution of ‘natural’ U isotope standard IRMM184 with sufficient ^233^U tracer to achieve a ^238^U/^233^U ratio of ~100 was prepared in a well-mixed batch and measured 125 times as the ‘IRMM84+1%^233^U’ reference solution. This was done to assess instrument performance, sensitivity and uncertainties of measurement.

**Chemical method of U extraction and purification.** The method was modified from [8-9] for separating and purifying U from urine. Batches of 24 samples consisted of 20 samples, two aliquots of the in-house large urine sample to which some IRMM184 (‘natural’ uranium of certified isotope composition) was added, and two blanks with 150ml of MQ H_2_0 instead of urine. Briefly, samples were weighed (150ml nominal sample), stirred on a hot plate at ~ 75^o^C, acidified with concentrated nitric acid; then ^233^U tracer, calcium nitrate and ammonium phosphate were added, further stirred, followed by neutralisation to pH 9 from ammonia addition that induced precipitation of calcium phosphate and allowed to settle. The precipitate was transferred to a pre-cleaned quartz beaker, re-dissolved in concentrated NHO_3_ and H_2_O_2_ and subjected to several cycles of evaporation at ~ 90°C and NHO_3_ and H_2_O_2_ until a pale or colourless salt. This was dissolved in 3ml of 1N AlNO_3_ - 3N HNO_3_ and loaded into columns containing 1.4ml UTEVA resin capped by 0.2ml of PFA resin (PFA is a resin that absorbs organic material), both precleaned prior to sample load with H_2_O, 0.6N HCl, and preconditioned in 3N HNO_3_. Alkali and non-actinide metal salts were eluted with 3N HNO3 and a modest amount of 6N HCl, and then uranium was removed by eluting with 0.6N HCl, evaporated with a small amount of concentrated HNO_3_ and then dissolved in 1.2 ml of 2% HNO_3_ for MC-ICP-MS mass spectrometry. The recovery of U from samples was likely to have averaged about 75%. For every ~20 samples processed, two blanks and two reference urine samples were processed. The blanks were processed identically to samples save for substituting purified H_2_O for urine. From these, the contamination introduced to the procedure (termed blank) was 11-38 *p*g, averaging 8-12 *p*g, but some samples contained even less uranium than blanks and so some adjustments were made to applicable blank amounts for some samples.

**Mass Spectrometry**. A Nu Instruments MC-ICP-MS instrument at the University of Portsmouth, equipped with multiple faraday and ion counting detectors was used for isotope analysis (^233^U, ^234^U, ^235^U, ^236^U, and^238^U). Sample solution was drawn into a Nu Instruments de-solvating nebuliser (DSN) via a Teflon or quartz nebuliser with a nominal uptake of 0.1ml/minute. At nominal flow rate, the instrument produced ~25M cps/ppb U. Occasionally samples were rerun when adequate sample remained after an analysis, sometimes diluting the sample with 2% HNO3 for additional full repeat measurement if the concentration was sufficient. Parameters of instrumental operation are listed in **Table S1**. The IRMM+1%^233^U reference solution was measured multiple times during every session to ensure quality control. The composition of this solution facilitated ion beam focussing and optimisation of intensity by the adjustment of DSN and mass spectrometer gas flows, torch position, accelerating potential, and focussing of numerous lenses, including quad settings for peak shape and multiple isotope collection peak alignment.

This protocol of measurement allowed all peaks to be measured in an IC simultaneously with ^238^U in a faraday detector (F); this allowed for the gain of each of the three multipliers to be measured using the IRMM+^233^U reference solution and compared to ^233^U(F)/^238^U(F) or ^235^U(F)/^238^U(F). The measurement of the 237 mass on an IC was designed to monitor the ‘tail’ arising from ion scattering from the large ^238^U peak as a function of pressure within the mass spectrometer. This ‘tailing’ arises from imperfect ion beam focussing and is a function of mass spectrometer design, compounded by collision of ions with occasional gas molecules/atoms, and causes broader peak shape, a narrower flat top of any given peak, and a tailing of lower energy U ions down-mass. The MC-ICP-MS design should result in a ‘tailing’ or mass abundance sensitivity of as little as 1-2 ppm of the ^238^U signal detected at mass 237, without any additional lenses to supress low energy ions. Because of pressure variations, the signal on mass 237 varied from 2-16 x 10^-6^ of that of 238, which required a correction procedure to take account of this temporal variation. This is an important effect because the study aimed to measure ^236^U/^238^U to a detection limit of ~1x10^-6^ to help confirm or refute the presence of non-natural uranium in conjunction with the ^235^U/^238^U ratio much like was done in.

Each sample measurement consisted of 5 cycles, each containing the 3 sequences, and took a total of about 8 minutes, consuming between 40-80% of the sample, depending upon the DSN uptake rate. The IRMM184+1%^233^U standard solution was measured approximately every 5 unknowns and is sufficiently concentrated to allow good precision measurements of all ratios while ensuring that no signals on any IC exceeded 10^6^ cps. The correction procedures for isotope ratio measurements are described below. Output from mass spectrometry measurements consisted of arithmetic means and their standard errors (standard deviation / √n) of intensities of the peaks on all masses and relevant isotope ratios using mass 238 as the usual denominator for isotope ratios.

Corrections to raw isotope data. Both samples and the IRMM184+1%^233^U standard solution were measured with the same mass spectrometric protocol. ^235^U(F)/^238^U(F) was measured and normalised to the IRMM184 certified ratio (0.0072623±0.0000033) after subtracting a negligible contribution from the ^233^U tracer to determine the mass bias, which was 0.726%/unit mass over the course of the study. Deadtime corrections of 20*n*s were then applied to all IC measurements. Next, the gains of each IC were determined by comparing a F/F ratio with its IC/F pair, for example ^235^U(F)/^238^U(F) of sequence 1 divided into the ^235^(IC)/^238^U(IC) of sequence 3. A time sequence of gains for each detector was determined for each session with samples using interpolated values. Where a value is the result of division or multiplication of other ratios, the uncertainties were propagated in quadrature, neglecting any small co-variance terms. Next, the contribution to ^236^U from the ‘tailing’ effect of ion scattering of ^238^U was done. This was facilitated by noting that a comparison of mass bias- and gain-corrected ratios of ^236^U(IC)/^238^U(F) of sequence 1 with 237(IC)/^238^U(F) of sequence 2 of the IRMM184+1%^233^U solution yields a highly linear array **(Fig. S1)**. The dispersion in 237(IC)/^238^U(F) in sequence 2, using the same IC for these two minor isotopes (and therefore reducing any uncertainty in IC gain) is a result of changes of pressure within the flight tube of the mass spectrometer, which varied during the course of the study and on a daily basis from 2 x 10^-8^ to 5 x 10^-9^ torr. The slope of this array is 2.995±0.050 (<2%) at the 95% confidence level, with an X-intercept of 1.95 x 10^-7^ ± 0.43 x 10^-7^, very close to the certified ^236^U/^238^U value of the IRMM184 standard (1.244 x 10^-7^), and closer still once the very small ^236^U contribution from the ^233^U tracer is taken into account.

The correction for tailing from ^238^U on mass 236 can be done two ways. Because all standards have a 237(IC)/^238^U(F) measurement, and following corrections for mass bias and gain, the 236 counts attributed to tailing are calculated by dividing the 237 counts by 2.995 and subtracting these from the total. When applied to the IRMM184+1%^233^U measurements, this allows a precision of ~1.5 x 10^-7^ on the final ^236^U/^238^U ratio for the IRMM184+1%^233^U measurement. After subtraction for the ^233^U tracer contribution, the mean ^236^U/^238^U was slightly different than the certified value though nearly overlapping within uncertainty. In addition, upon measurement of 2% HNO3 solutions and in conjunction with this slight difference (~0.2 x 10^-7^) from the ^236^U/^238^U certified value (1.22 x 10^-7^) we noted excess counts on mass 236; we had measured 1-5 cps on mass 236 when measuring 2% HNO_3_ solution and so a 2.5cps subtraction on the ^236^U measurement for all samples and standards was done; this brought the mean measured value of ^236^U/^238^U into coincidence with the certified value, the former being 1.24 ± 1.4 x 10^-7^. The ‘noise’ or limit of detection of this correction is approximately the dispersion in ^236^U/^238^U at any given 237/^238^U value, or about ±1.5 x 10^-7^, but it is also affected by the ~2% uncertainty in the slope of the line. The dispersion in ^236^U/^238^U corresponds to approximately 2-13 cps, depending upon pressure and therefore magnitude of 237/238 measurement. This additional uncertainty has been taken into account for all analyses by applying a ± 5% uncertainty to the slope of the correction and in addition adding ±2 cps uncertainty to the ^236^U/^238^U ratio after this correction has been applied.

The final data for samples and in-house urine samples, and from the IRMM184+1%^233^U solution are shown in **tables S3 and S4**, respectively. Once all of these corrections are made, the analyses of the IRMM184 reference solution, yielded ^234^U/^238^U, ^235^U/^238^U and ^236^U/^238^U values of 5.344 x 10^-5^ ± 2.0 x 10^-6^, 0.0072614 ± 0.0000025 and 1.1 x 10^-7^ ±0.1 x 10^-7^, respectively, within the certified value of the IRMM184. The ^234^U/^238^U of urine samples is considerable and reflects the natural variation observed in water that has interacted with uranium within minerals, rocks and soil [10]; its natural variability therefore prevents it being used as a proxy for detection of DU.

**Figure S2** shows a linearized probability plot of ^236^U/^238^U in the IRMM184+1%^233^U solution and indicates that although generally there is excellent coherence of the measurements, there is a tendency of excess counts on mass 236 to be present in a small minority of the IRMM184+1%^233^U measurements, which raises the ^236^U/^238^U isotope value in some measurements. This is likely to be the same type of interference as is described below for samples, though very much less prevalent, with a magnitude of excess counts of up to 3cps. The above method of tailing correction is reliable for uranium reference solutions but is not reliable for urine samples.

A second correction method for tailing from ^238^U on mass 236, applicable to samples, is to derive a correction from ^238^U on the ^236^U by using the value of correction to ^236^U/^238^U of IRMM184+1%^233^U solution that were measured alongside groups of samples; this allows one to take into account any time-dependent variations in tailing during the daily measurement session. This is essential for urine samples because of the variable presence of stray excess counts on the 237 mass, a real effect that is probably produced by a charged organic molecule with a mass of ~ 236.8. This anomaly has been observed in mass scans with the offending excess 237 peak slightly offset from the nominal 237 mass, as shown in **figure S3**. The molecule(s) has not been identified, but it appears to be present in measurable quantities only in samples and is likely related to incomplete removal of all traces of organic matter not generally present in standard analyses.

**Figure S4** shows mass bias- and gain-corrected measurements of ^236^U(IC)/^238^U(F) v. 237(IC)/^238^U(F) of urine samples and IRMM184 reference solutions. If compared to **figure S1**, there is a great deal more scatter, mainly in excess 237 counts, but a potential lesser magnitude additional contribution from excess 236 counts. The magnitude of excess counts is far higher than the counts arising from the tailing effect. The highly correlated array for standards is also included in **figure S4** as the linear lower right bound of the cluster of data) defines the lower limit of sample analyses, with only a few measurements falling below this bounding array of reference solution data.

The excess scatter is a result of the presumed organic ion at mass 236.8 adding stray counts to the measured 237 signal. The actual magnitude of the excess 237 varied from 0-200 cps, and for 236 0-~20 cps. For urine samples containing no ^236^U, 3 standard deviations of the 236 mass was 11 cps; as a result, no analyses can be considered reliably measured that have <11 cps on 236 mass. Samples with either the 95% confidence measurement uncertainty exceeding the ^236^U/^238^U value or being <11 cps are regarded as below the limit of detection (LOD) as shown in **table S3**. Only 6 measurements of the dataset had measurements above the LOD (**Fig. S5a**) and these had a far higher measured ^236^U/^238^U than should be expected given the maximum 95% confidence value of ^238^U/^235^U value (**Fig.S5b**), and as such, are likely caused by higher excess 236 counts than the cut-off for LOD. The trajectory indicates that the higher ^236^U/^238^U is an artefact of organic molecule interference and not DU.

**Statistical aspects.**

The study cites geometric means for all data, appropriate for skewed distributions, and where appropriate, asymmetric uncertainties arising from this are quoted. P-values of the *t*-test were calculated in the standard way using Excel functions using measured values for sub-groups, assuming a two-tailed distribution with similar variances. Where a Kruskal-Wallis test is cited, this has been calculated using an Excel add-in from the Real Statistics Resource Pack software (Release 6.8) of Charles Zaiontz ([www.real-statistics.com](http://www.real-statistics.com)).

**References cited in Supplemental Material**

1. Royal Society. *The health hazards of depleted uranium munitions: Part I* (2001).

2. WHO (World Health Organisation). Depleted uranium: Sources, Exposure and Health Effects. Geneva World Health Organization <https://apps.who.int/iris/handle/10665/66930> (2001).

3. Etherington, G. The solubility of inhaled DU and its influence on urine excretion. Depleted Uranium Oversight Board, https://drive.google.com/file/d/1Z56XXc51IAUw8qWwMetPOIhPedLWBuVO/view?usp=sharing (2004).

4. Bair, W. J. et al. Human respiratory tract model for radiological protection. A report of a Task Group of the International Commission on Radiological Protection. *Ann ICRP.* **24,** 1-482 (1994).

5. Parkhurst, M. A. et al. Capstone Depleted Uranium Aerosols: Generation and Characterization, *Pacific Northwest National Laboratory*-14168 https://www.pnnl.gov/main/publications/external/technical_reports/PNNL-14168.pdf (2004).

6. Lloyd, N. S. et al. The morphologies and compositions of depleted uranium particles from an environmental case-study. *Mineralogical Magazine.* **73**, 495-510 (2009).

7. Dorsey, C. D., Engelhardt, S. M., Squibb, K. S. & McDiarmid, M. A. Biological monitoring for depleted uranium exposure in U.S. Veterans. *Environ Health Perspect.* **117**, 953-956 (2009).

8. Parrish, R. R. et al. Determination of MC-ICP-MS 238U/235U, 236U/238U and uranium concentration in urine using SF-ICP-MS and MC-ICP-MS: an interlaboratory comparison. *Health Phys.* **90**, 127-138 (2006).

9. Parrish, R. R. et al. Depleted uranium contamination by inhalation exposure and its detection after approximately 20 years: implications for human health assessment. *Sci Total Environ.* **390,** 58-68 (2008).

10. Ivanovich, M. & Harmon, R. S. *Uranium-Series Disequilibrium: Applications to Earth, Marine, and Environmental Sciences*. 2^nd^ edition. UK Clarendon Press (1992).

**Captions for Supplemental figures.**

**Figure S1.** Plot of signal ratios of 236/238 v. 237/238 in the IRMM184+1%233U measurements illustrating the strong correlation between 237 and 236, a result of tailing from the much larger ^238^U isotope.

**Figure S2.** Linearized probability plot of final ^236^U/^238^U measurements on the IRMM184+1%^233^U solution showing predominantly Gaussian behaviour of measurements aside from higher values beyond the 95th percentile that have excess 236 counts.

**Figure S3.** Screen capture mass scan of ^238^U (faraday detector, top scan), ^235^U (ion counting detector, middle scan), and 237 mass (ion counting, lower scan offset to lower mass at about 236.8.

**Figure S4.** Plot of 237/238 v. 236/238 to illustrate the scatter towards excess 236 and 237 counts above the diagonally bounding linear array of constant 236/237 slope from that of IRMM184 standards (see **Fig S1**).

**Figure S5.** Plot of rank order of samples with ^236^U signal > 0.5cps and ^236^U/^238^U v. ^235^U/^238^U for the 6 samples with ^236^U/^238^U above LOD of 11 cps. Lines of mixing of DU and NU, and of the trajectory of excess 236 counts are shown.

**Table S1. ICP-MC-MS operating conditions**

| Category | Condition |
| --- | --- |
| ICP-MS Instrument | Nu Instruments NuPlasma ICP-MC-MS, at University of Portsmouth |
| Sample introduction | Nu Instruments DSN aerosol with membrane Ar gas flow 2.8l/m, nebulizer pressure 30 |
| RF power (W) | 1300W |
| Carrier gas (L/min) | Ar = 0.87 L/min |
| Masses measured | 233, 234, 235, 236, 237, 238 |
| Measurement time in each sequence | 10s followed by 2s magnet settle after each mass shift to measure the 3 sequences; 5 measurements of this series of sequences for each final analysis |
| Measurement mode | 3 sequences of multicollection, see **Table S2**. |
| Calibration strategy | IRMM184+1%^233^U solution, natural composition of uranium, for peak centring, peak shape optimization, sensitivity, etc. (IRMM, Institute of Reference Materials and Measurements, Geel, Belgium) |
| Data processing package used / | Bespoke Excel spreadsheet with corrections for mass bias using IRMM 235/238, gain of ion counters, hydride and mass abundance sensitivity, tailing using 237 mass |
| Quality control / Validation | 125 measurements of IRMM184+1%^233^U solution, 14 measurements of in-house urine sample to which was added variable amounts of IRMM184+1%^233^U solution. |

During a run, there was initial peak centring, followed by measurement of isotope signals across multiple detectors in three sequences with a shift of one mass unit (1 Dalton) into any given collector. This facilitated measurement in either faraday cups (F) or ion counting electron multipliers (IC) in sequence 1 of ^238^U(F), ^235^U(F), ^233^U(F), ^236^U(IC), and ^234^U(IC); in sequence 2 of ^238^U(F), 237 mass (IC), ^235^U(IC), ^233^U (IC), ^236^U(IC); and finally in sequence 3 of ^238^U(F), ^235^U(IC), and ^233^U(IC). This series of peak shifts in the multiple collector array is shown in **Table S2**.

**Table S2. ICP-MC-MS collector array**

| **Magnet sequence** | **IC2** | **L4 far** | **IC1** | **L3 far** | **IC0** | **L2 far** | **L1 far** | **Ax far** |
| --- | --- | --- | --- | --- | --- | --- | --- | --- |
| **Seq 1** |  | **233** | **234** | **235** | **236** |  | **238** |  |
| **Seq 2** | **233** |  | **235** |  | **237** | **238** |  |  |
| **Seq 3** |  |  | **233** |  | **235** |  |  | **238** |

*Note: far, faraday cup; IC, ion counter; 238, mass used for peak centering*

**Tables S3 and S4** are tabulated pdf files of the full data for individual analyses of samples and the IRMM84 reference solution and are submitted as separate files.

**Figure S1.**

**Figure S1.** Plot of signal ratios of 236/238 v. 237/238 in the IRMM184+1%^233^U measurements illustrating the strong correlation between 237 and 236, a result of tailing from the much larger ^238^U isotope.

**Figure S2.**

**Figure S2.** Linearized probability plot of final ^236^U/^238^U measurements on the IRMM184+1%^233^U solution showing predominantly Gaussian behaviour of measurements aside from higher values beyond the 95^th^ percentile that have excess 236 counts.

**Figure S3.**


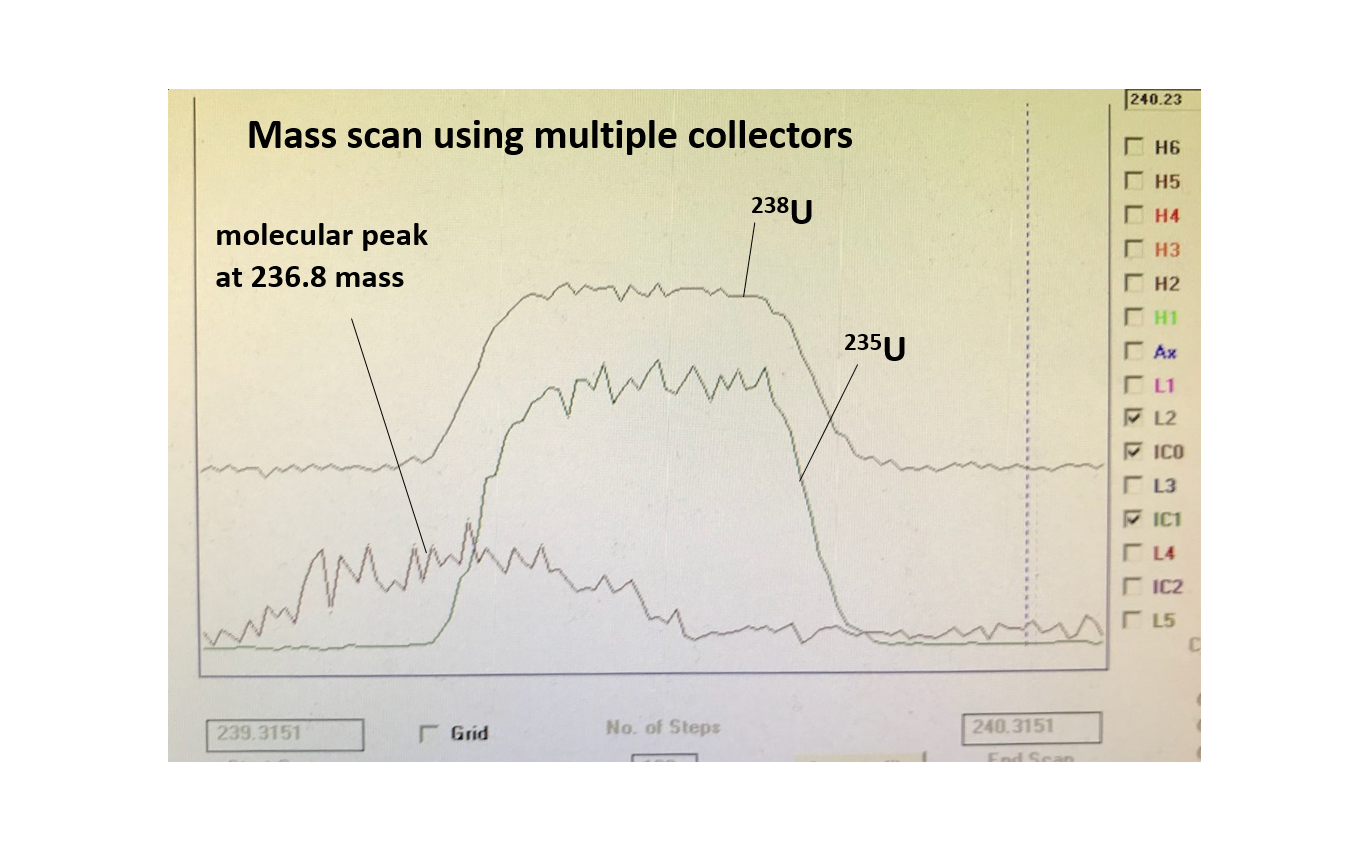


**Figure S3.** Screen capture mass scan of ^238^U (faraday detector, top scan), ^235^U (ion counting detector, middle scan), and 237 mass (ion counting, lower scan offset to lower mass at about 236.8.

**Figure S4.**

**Figure S4.** Plot of 237/238 v. 236/238 to illustrate the scatter towards excess 236 and 237 counts above the diagonally bounding linear array of constant 236/237 slope from that of IRMM184 standards (see **Fig S1**).

**Figure S5.**


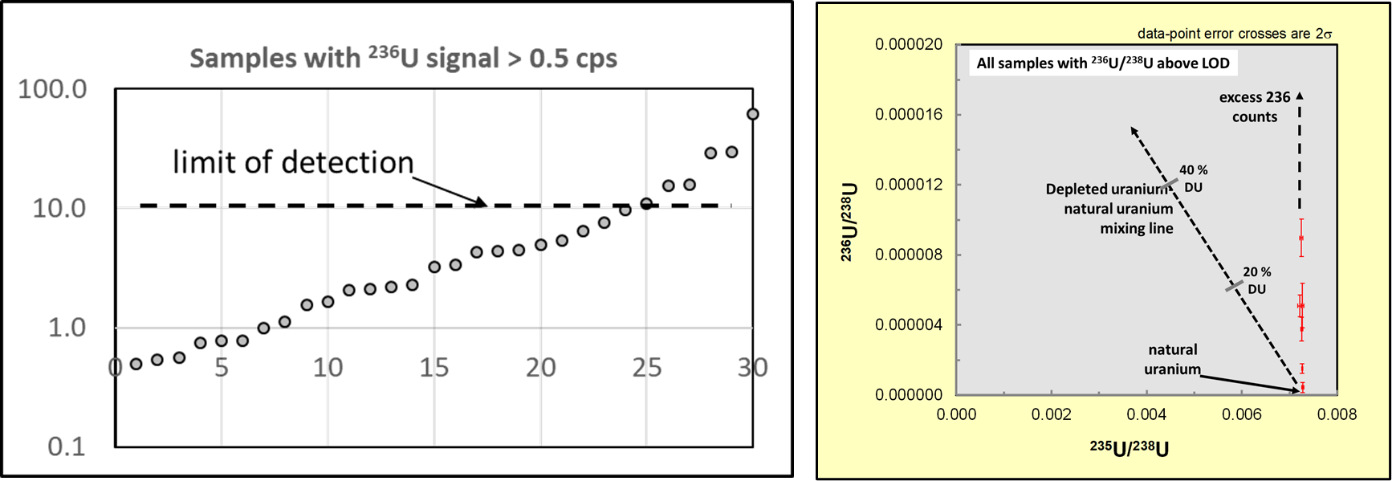


**Figure S5.** Plot of rank order of samples with ^236^U signal > 0.5cps and ^236^U/^238^U v. ^235^U/^238^U for the 6 samples with ^236^U/^238^U above LOD of 11 cps. Lines of mixing of DU and NU, and of the trajectory of excess 236 counts are shown.
