# Supplementary material for: Resolving whether inhalation of depleted uranium contributed to Gulf War Illness using high-sensitivity mass spectrometry
Source: Sci Rep. 2021 Feb 18;11:3218. doi: 10.1038/s41598-021-82535-3 (PMC7893152; doi:10.1038/s41598-021-82535-3)
Supplement: Supplementary file 2 — Supplementary Information 2. [file 41598_2021_82535_MOESM2_ESM.pdf]

Table S3. U isotope measurements of samples

Sample Name	^{238}U *n* g/kg	24h U excretion	*n* g U/g creatinine	^{238}U intensity (cps)	^{234}U / ^{238}U	±	^{235}U / ^{238}U	±	^{236}U / ^{238}U	±	^{238}U / ^{235}U	± 2sd	InNI study[##]	Group [φφ]	DU Level [$$]
Study Cohort samples															
GWS 0041	3.1	6.19	2.89	1.49E+07	6.44E-05	6.1E-07	7.29E-03	1.6E-05	<LOD		137.1	0.6	2	-1	4
GWN-0074	0.8	2.02	0.85	9.48E+05	9.17E-05	3.8E-06	7.29E-03	4.7E-05	<LOD		137.1	1.8	3	-1	4
GWN-0067	0.3	0.65	0.23	2.58E+05	9.63E-05	5.5E-06	7.32E-03	6.9E-05	<LOD		136.5	2.6	3	-1	4
GWN-0071	0.2	0.24	0.19	2.19E+05	7.12E-05	1.1E-05	7.36E-03	1.1E-04	<LOD		135.9	4.0	3	-1	4
GWN-0069	0.5	0.83	0.39	1.01E+06	8.96E-05	3.7E-06	7.29E-03	5.5E-05	<LOD		137.2	2.1	3	-1	4
GWN-0065	2.2	10.43	5.83	1.34E+06	9.16E-05	2.7E-06	7.28E-03	5.6E-05	<LOD		137.3	2.1	3	-1	4
GWS 0036	0.8	1.27	0.87	4.90E+06	6.17E-05	2.8E-06	7.27E-03	2.6E-05	<LOD		137.5	1.0	2	-1	4
GWN-0076	3.8	8.08	7.15	6.78E+06	6.14E-05	2.3E-06	7.27E-03	3.7E-05	<LOD		137.6	1.4	3	-1	4
GWS-0017	4.3	11.22	7.38	7.48E+06	1.03E-04	9.5E-07	7.26E-03	2.4E-05	<LOD		137.8	0.9	2	-1	4
GWN-0063	0.5	0.75	0.25	3.42E+05	1.09E-04	3.1E-06	7.26E-03	6.4E-05	<LOD		137.8	2.4	3	-1	4
GWN-0066	1.6	2.47	0.97	2.24E+06	6.84E-05	2.0E-06	7.26E-03	3.4E-05	<LOD		137.8	1.3	3	-1	4
GWN-0087	2.0	3.48	1.98	4.21E+06	1.05E-04	1.9E-06	7.26E-03	2.1E-05	3.8E-06	6.8E-07	137.8	0.8	3	-1	4
GWN-0092	0.9	1.47	0.75	1.82E+06	6.58E-05	3.5E-06	7.25E-03	3.9E-05	<LOD		137.9	1.5	3	-1	
GWN-0073	0.8	2.73	1.63	1.39E+06	6.65E-05	3.9E-06	7.25E-03	4.9E-05	<LOD		137.9	1.9	3	-1	4
GWN-0059	3.0	9.76	4.38	3.87E+06	1.50E-04	2.5E-06	7.25E-03	2.8E-05	<LOD		138.0	1.1	3	-1	4
GWS-0049	4.0	5.99	2.67	2.75E+06	6.43E-05	1.3E-06	7.25E-03	2.6E-05	<LOD		138.0	1.0	2	-1	4
GWN-0085	13.4	30.16	10.81	1.51E+07	6.75E-05	2.2E-06	7.25E-03	3.6E-05	<LOD		138.0	1.4	3	-1	4
GWN-0061	0.6	1.94	1.25	7.42E+05	8.95E-05	3.6E-06	7.25E-03	5.1E-05	<LOD		138.0	1.9	3	-1	4
GWN-0064	0.8	2.53	2.13	8.11E+05	7.76E-05	3.8E-06	7.24E-03	3.6E-05	<LOD		138.1	1.4	3	-1	4
GWS-0014	0.5	1.03	0.47	8.92E+05	8.02E-05	3.8E-06	7.22E-03	4.2E-05	<LOD		138.4	1.6	2	-1	4
GWS-0029	0.1	0.17	0.09	1.22E+05	7.97E-05	1.5E-05	7.22E-03	2.3E-04	<LOD		138.4	8.8	2	-1	4
GWN-0014	0.2	0.32	0.14	3.78E+05	9.83E-05	6.9E-06	7.36E-03	6.1E-05	<LOD		135.9	2.3	3	0	4
GWN-0048	0.4	0.71	0.30	5.88E+05	8.76E-05	4.4E-06	7.34E-03	6.7E-05	<LOD		136.2	2.5	3	0	4
GWN-0070	3.4	8.93	1.96	2.01E+06	6.72E-05	2.4E-06	7.29E-03	3.7E-05	<LOD		137.2	1.4	3	0	4
GWN-0023	2.1	6.13	3.46	1.56E+06	6.34E-05	1.2E-06	7.28E-03	2.9E-05	<LOD		137.4	1.1	3	0	4
GWN-0037	6.8	11.86	6.11	5.24E+06	6.21E-05	8.6E-07	7.28E-03	3.1E-05	<LOD		137.4	1.2	3	0	4
GWS-0019	0.9	2.56	1.02	2.02E+06	8.54E-05	2.1E-06	7.28E-03	2.8E-05	<LOD		137.4	1.1	2	0	4
GWS-0003	63.2	96.43	54.48	3.58E+07	8.58E-05	3.5E-07	7.28E-03	1.9E-05	4.3E-07	3.0E-07	137.4	0.7	1	0	
GWS-0005	1.9	4.06	2.90	1.23E+06	7.61E-05	1.6E-06	7.27E-03	1.4E-05	<LOD		137.5	0.5	2	0	4
GWS 0035	20.7	63.39	35.02	4.12E+07	5.41E-05	4.5E-07	7.27E-03	9.4E-06	1.5E-06	2.8E-07	137.6	0.4	2	0	4
GWS-0023	3.3	3.87	2.49	4.37E+06	7.33E-05	1.4E-06	7.27E-03	3.1E-05	<LOD		137.6	1.2	2	0	
GWS-0006	1.7	3.73	2.16	1.58E+06	6.89E-05	1.8E-06	7.26E-03	1.4E-05	<LOD		137.7	0.5	2	0	4
GWN-0078	0.4	0.43	0.19	4.09E+05	9.34E-05	6.0E-06	7.26E-03	7.9E-05	<LOD		137.7	3.0	3	0	4
GWN-0055	1.7	3.22	1.77	3.38E+06	7.45E-05	1.7E-06	7.26E-03	3.4E-05	<LOD		137.7	1.3	3	0	3
GWN-0041	2.6	7.90	5.25	5.52E+06	6.27E-05	1.3E-06	7.25E-03	2.3E-05	<LOD		137.8	0.9	3	0	4
GWN-0020	13.0	19.49	11.36	2.08E+07	6.29E-05	6.4E-07	7.25E-03	3.3E-05	<LOD		137.9	1.3	3	0	4
GWN-0052	2.1	6.60	2.47	2.71E+06	1.04E-04	1.4E-06	7.25E-03	2.9E-05	<LOD		137.9	1.1	3	0	4
GWN-0050	4.9	13.60	6.07	7.29E+06	7.01E-05	9.1E-07	7.25E-03	3.1E-05	<LOD		137.9	1.2	3	0	1
GWS-0058	2.7	9.33	4.91	5.21E+05	6.70E-05	1.8E-06	7.24E-03	3.1E-05	<LOD		138.1	1.2	2	0	4
GWN-0060	1.5	2.76	1.61	1.67E+06	6.75E-05	2.3E-06	7.24E-03	3.3E-05	<LOD		138.1	1.3	3	0	3
GWN-0053	11.6	15.65	5.92	2.18E+07	6.29E-05	6.3E-07	7.24E-03	3.4E-05	<LOD		138.1	1.3	3	0	2
GWS-0011	0.2	0.76	0.37	5.32E+05	8.66E-05	6.5E-06	7.24E-03	6.9E-05	<LOD		138.1	2.6	2	0	4
GWN-0046	2.4	2.96	1.51	2.83E+06	7.33E-05	1.5E-06	7.24E-03	3.1E-05	<LOD		138.2	1.2	3	0	4
GWS-0015	0.7	1.14	0.78	1.60E+06	7.37E-05	2.9E-06	7.23E-03	3.7E-05	<LOD		138.3	1.4	2	0	4
GWS-0030	2.7	3.97	2.17	3.22E+06	6.88E-05	2.4E-06	7.22E-03	4.2E-05	<LOD		138.4	1.6	2	0	4
GWS 0028	0.4	1.16	0.45	1.74E+06	6.97E-05	5.0E-06	7.22E-03	4.8E-05	<LOD		138.4	1.8	2	0	3

Table S3. U isotope measurements of samples

Sample Name	^{238}U *n* g/kg	24h U excretion	*n* g U/g creatinine	^{238}U intensity (cps)	^{234}U / ^{238}U	±	^{235}U / ^{238}U	±	^{236}U / ^{238}U	±	^{238}U / ^{235}U	± 2sd	InNI study##	Group ϕϕ	DU Level $$
GWN-0042	0.5	0.85	0.40	8.29E+05	8.01E-05	4.3E-06	7.23E-03	6.7E-05	<LOD		138.4	2.6	3	0	4
GWN-0051	0.3	0.41	0.18	2.81E+05	1.01E-04	5.0E-06	7.18E-03	1.1E-04	<LOD		139.2	4.1	3	0	4
GWN-0035	1.1	1.20	0.86	1.48E+06	7.94E-05	2.3E-06	7.29E-03	3.2E-05	<LOD		137.1	1.2	3	1	1
GWN-0031	1.0	1.38	0.85	1.03E+06	7.15E-05	1.9E-06	7.30E-03	3.3E-05	<LOD		137.0	1.2	3	1	3
GWN-0005	1.6	3.93	1.97	2.68E+06	6.82E-05	1.8E-06	7.29E-03	3.6E-05	<LOD		137.2	1.4	3	1	4
GWS-0026	0.3	1.64	0.69	4.09E+05	7.56E-05	1.3E-05	7.29E-03	5.1E-05	<LOD		137.1	1.9	2	1	4
GWS-0027	0.3	0.64	0.30	1.79E+05	6.42E-05	9.5E-06	7.33E-03	8.7E-05	<LOD		136.4	3.2	2	1	4
GWN-0089	0.2	0.22	0.13	3.79E+05	6.73E-05	1.2E-05	7.32E-03	1.2E-04	<LOD		136.6	4.4	3	1	4
GWN-0007	1.4	1.45	1.07	2.18E+06	7.12E-05	1.6E-06	7.29E-03	3.4E-05	<LOD		137.2	1.3	3	1	1
GWS-0053	1.3	4.11	1.28	1.22E+06	6.36E-05	1.8E-06	7.28E-03	2.4E-05	<LOD		137.3	0.9	2	1	3
GWN-0006	1.1	2.96	1.80	7.52E+05	8.34E-05	4.2E-06	7.28E-03	3.4E-05	<LOD		137.3	1.3	3	1	3
GWS-0054	0.8	2.38	0.97	8.45E+05	6.51E-05	2.7E-06	7.28E-03	3.6E-05	<LOD		137.4	1.4	2	1	4
GWN-0029	3.0	8.27	8.99	2.91E+06	6.56E-05	1.1E-06	7.28E-03	3.0E-05	<LOD		137.4	1.1	3	1	3
GWS-0046	1.4	3.21	1.43	6.48E+05	6.36E-05	3.0E-06	7.28E-03	3.4E-05	<LOD		137.4	1.3	2	1	4
GWN-0056	1.2	1.79	1.38	1.50E+06	7.22E-05	2.4E-06	7.28E-03	3.4E-05	<LOD		137.5	1.3	3	1	4
GWN-0034	6.1	5.52	4.70	1.03E+07	8.29E-05	1.0E-06	7.27E-03	2.9E-05	<LOD		137.5	1.1	3	1	4
GWN-0044	0.8	1.10	0.62	1.46E+06	7.50E-05	2.8E-06	7.27E-03	4.1E-05	<LOD		137.5	1.5	3	1	2
GWN-0009	2.5	3.65	2.91	3.15E+06	1.61E-04	2.6E-06	7.27E-03	3.4E-05	<LOD		137.5	1.3	3	1	4
GWS-0055	0.3	0.52	0.19	2.68E+05	7.34E-05	5.5E-06	7.27E-03	5.1E-05	<LOD		137.5	1.9	2	1	2
GWN-0049	4.6	13.26	6.38	7.46E+06	6.67E-05	9.5E-07	7.27E-03	2.8E-05	<LOD		137.5	1.0	3	1	3
GWN-0054	0.5	0.88	0.54	1.14E+06	7.36E-05	4.2E-06	7.27E-03	5.1E-05	<LOD		137.5	1.9	3	1	4
GWS-0045	3.4	7.28	2.84	1.01E+07	6.02E-05	1.0E-06	7.27E-03	1.3E-05	<LOD		137.6	0.5	2	1	4
GWS 0022	2.4	8.76	3.51	3.82E+06	7.03E-05	1.1E-06	7.27E-03	1.6E-05	<LOD		137.6	0.6	2	1	4
GWS-0050	4.2	5.71	4.08	5.49E+06	5.87E-05	1.1E-06	7.26E-03	2.1E-05	<LOD		137.7	0.8	2	1	4
GWN-0096	1.1	1.41	1.47	1.16E+06	6.27E-05	2.5E-06	7.26E-03	3.8E-05	<LOD		137.7	1.4	3	1	4
GWN-0039	1.6	4.68	2.29	1.68E+06	7.81E-05	1.6E-06	7.26E-03	2.9E-05	<LOD		137.7	1.1	3	1	2
GWN-0081	1.9	2.32	1.35	2.71E+06	7.12E-05	3.1E-06	7.26E-03	4.4E-05	<LOD		137.8	1.7	3	1	1
GWN-0093	1.7	6.50	3.56	1.90E+06	7.15E-05	2.1E-06	7.25E-03	2.4E-05	<LOD		137.9	0.9	3	1	4
GWN-0088	1.1	1.27	0.57	1.64E+06	6.72E-05	2.5E-06	7.25E-03	3.2E-05	<LOD		137.9	1.2	3	1	3
GWS-0034	6.7	12.55	7.38	5.54E+06	7.72E-05	1.4E-06	7.25E-03	4.0E-05	<LOD		137.9	1.5	2	1	4
GWN-0011	2.7	5.57	2.85	6.62E+06	7.20E-05	1.4E-06	7.22E-03	3.5E-05	<LOD		138.5	1.3	3	1	4
GWN-0082	0.8	2.19	1.27	1.38E+06	1.01E-04	4.1E-06	7.23E-03	4.9E-05	<LOD		138.4	1.9	3	1	4
GWN-0080	0.6	1.05	0.42	4.71E+05	7.37E-05	7.8E-06	7.23E-03	5.1E-05	<LOD		138.2	2.0	3	1	1
GWN-0003	0.9	1.84	1.05	8.82E+05	1.14E-04	3.0E-06	7.31E-03	3.5E-05	<LOD		136.8	1.3	3	2	4
GWN-0045	0.9	1.60	0.98	1.84E+06	7.71E-05	2.7E-06	7.33E-03	3.7E-05	<LOD		136.4	1.4	3	2	4
GWN-0008	0.5	3.01	1.94	1.13E+06	7.28E-05	3.1E-06	7.29E-03	3.8E-05	<LOD		137.1	1.4	3	2	4
GWN-0086	0.5	1.17	0.44	9.13E+05	7.50E-05	3.9E-06	7.30E-03	4.6E-05	<LOD		137.0	1.7	3	2	4
GWN-0028	3.5	4.14	1.87	1.81E+06	8.84E-05	3.6E-06	7.29E-03	3.2E-05	<LOD		137.2	1.2	3	2	4
GWN-0047	0.8	2.40	1.34	1.13E+06	9.32E-05	2.6E-06	7.29E-03	6.4E-05	<LOD		137.3	2.4	3	2	4
GWS-0044	1.0	2.77	1.54	1.97E+06	8.40E-05	2.0E-06	7.28E-03	1.5E-05	<LOD		137.3	0.6	2	2	3
GWN-0021	12.7	18.95	16.63	1.07E+07	6.94E-05	7.7E-07	7.28E-03	3.4E-05	<LOD		137.3	1.3	3	2	3
GWS-0020	5.4	5.70	3.17	7.34E+05	8.87E-05	1.8E-06	7.28E-03	3.1E-05	<LOD		137.4	1.2	2	2	3
GWN-0024	2.1	4.69	2.02	1.52E+06	6.78E-05	1.5E-06	7.28E-03	3.1E-05	<LOD		137.4	1.2	3	2	2
GWN-0072	0.4	2.15	0.70	8.38E+05	6.54E-05	6.1E-06	7.28E-03	6.7E-05	<LOD		137.4	2.5	3	2	4
GWS-0059	2.7	8.60	4.39	3.47E+06	5.85E-05	1.2E-06	7.28E-03	2.3E-05	<LOD		137.4	0.9	2	2	4
GWS-0004	1.3	3.69	2.27	1.65E+06	6.59E-05	1.9E-06	7.28E-03	2.6E-05	<LOD		137.4	1.0	2	2	2
GWN-0057	8.2	28.76	23.87	1.65E+07	9.53E-05	4.7E-07	7.28E-03	1.1E-05	<LOD		137.4	0.4	3	2	3
GWN-0025	13.6	6.82	5.41	1.73E+07	6.65E-05	8.2E-07	7.28E-03	2.9E-05	<LOD		137.4	1.1	3	2	4

Table S3. U isotope measurements of samples

Sample Name	^{238}U *n* g/kg	24h U excretion	*n* g U/g creatinine	^{238}U intensity (cps)	^{234}U / ^{238}U	±	^{235}U / ^{238}U	±	^{236}U / ^{238}U	±	^{238}U / ^{235}U	± 2sd	InNI study##	Group φφ	DU Level $$
GWN-0036	4.1	3.76	2.40	5.89E+06	6.87E-05	8.5E-07	7.27E-03	3.3E-05	<LOD		137.5	1.3	3	2	4
GWS-0009	1.6	3.45	1.38	2.15E+06	9.65E-05	1.9E-06	7.27E-03	2.7E-05	5.1E-06	1.3E-06	137.5	1.0	2	2	4
GWN-0097	6.7	16.80	9.08	1.40E+06	6.10E-05	3.2E-06	7.27E-03	4.6E-05	<LOD		137.5	1.7	3	2	4
GWS-0060	3.0	9.19	3.08	3.29E+06	8.28E-05	1.4E-06	7.27E-03	2.4E-05	<LOD		137.5	0.9	2	2	4
GWS-0057	10.7	21.86	13.75	1.90E+07	5.44E-05	7.9E-07	7.27E-03	2.2E-05	<LOD		137.5	0.8	2	2	4
GWN-0026	3.2	6.96	2.6	1677008.3	7.72E-05	1.8E-06	7.27E-03	3.5E-05	<LOD		137.6	1.3	3	2	3
GWN-0027	2.1	4.74	2.37	1.94E+06	9.22E-05	2.2E-06	7.27E-03	4.0E-05	<LOD		137.6	1.5	3	2	1
GWN-0062	16.3	26.52	6.96	3.93E+07	6.82E-05	8.1E-07	7.27E-03	2.6E-05	<LOD		137.6	1.0	3	2	3
GWS-0013	1.7	2.86	1.68	2.96E+06	7.74E-05	1.3E-06	7.26E-03	2.5E-05	<LOD		137.7	0.9	2	2	4
GWS-0016	3.0	7.12	4.60	3.31E+06	7.45E-05	9.8E-07	7.26E-03	2.3E-05	<LOD		137.7	0.9	2	2	3
GWN-0018	3.3	9.26	4.04	4.60E+06	6.62E-05	1.3E-06	7.26E-03	3.6E-05	<LOD		137.7	1.4	3	2	2
GWN-0013	1.1	2.09	1.04	2.67E+06	7.96E-05	1.7E-06	7.26E-03	3.6E-05	<LOD		137.8	1.4	3	2	2
GWS-0012	0.7	2.29	0.62	8.55E+05	7.28E-05	2.7E-06	7.26E-03	3.4E-05	<LOD		137.8	1.3	2	2	2
GWN-0079	2.8	7.77	4.56	4.07E+06	6.32E-05	2.3E-06	7.25E-03	3.9E-05	<LOD		137.9	1.5	3	2	2
GWN-0094	0.9	0.74	0.55	1.37E+06	7.03E-05	3.1E-06	7.25E-03	3.6E-05	<LOD		137.9	1.4	3	2	4
GWN-0012	0.9	1.39	0.84	1.32E+06	7.33E-05	2.2E-06	7.25E-03	3.7E-05	<LOD		137.9	1.4	3	2	3
GWS-0007	1.3	3.57	2.09	4.97E+05	9.01E-05	3.8E-06	7.24E-03	2.7E-05	<LOD		138.0	1.0	2	2	4
GWN-0095	3.3	6.28	4.52	2.33E+06	5.53E-05	1.4E-06	7.24E-03	2.9E-05	<LOD		138.0	1.1	3	2	4
GWS-0031	8.9	16.52	7.13	9.72E+05	6.60E-05	2.6E-06	7.24E-03	3.3E-05	<LOD		138.1	1.3	2	2	3
GWS 0032	0.5	2.00	0.92	4.36E+06	6.56E-05	3.9E-06	7.24E-03	3.5E-05	<LOD		138.1	1.3	2	2	4
GWS-0010	0.4	0.76	0.58	7.91E+05	8.25E-05	4.1E-06	7.24E-03	4.4E-05	<LOD		138.1	1.7	2	2	4
GWS-0018	0.6	0.70	0.88	1.12E+06	7.83E-05	2.9E-06	7.24E-03	4.3E-05	<LOD		138.1	1.7	2	2	4
GWS-0025	2.1	5.23	2.50	6.14E+05	6.97E-05	2.9E-06	7.23E-03	3.1E-05	<LOD		138.3	1.2	2	2	3
GWN-0084	2.3	5.86	3.66	3.87E+06	9.25E-05	3.1E-06	7.23E-03	3.8E-05	<LOD		138.2	1.4	3	2	4
GWN-0090	1.5	3.56	1.75	1.47E+06	7.28E-05	3.3E-06	7.22E-03	4.4E-05	<LOD		138.5	1.7	3	2	3
GWS 0037	0.2	0.90	0.46	1.17E+06	1.29E-04	2.2E-06	7.21E-03	7.0E-05	<LOD		138.6	2.7	2	2	3
GWS-0008	0.2	0.94	0.51	5.05E+05	1.36E-04	6.3E-06	7.15E-03	7.7E-05	<LOD		139.8	3.0	2	2	4
GWS-0043	2.2	7.73	4.86	4.93E+06	6.45E-05	1.3E-06	7.30E-03	1.5E-05	<LOD		137.0	0.6	2	3	4
GWS 0040	2.5	4.73	3.67	1.18E+07	6.41E-05	7.2E-07	7.29E-03	1.7E-05	<LOD		137.1	0.6	2	3	4
GWN-0038	1.2	3.15	1.95	1.06E+06	8.12E-05	2.1E-06	7.31E-03	3.1E-05	<LOD		136.7	1.1	3	3	4
GWS-0051	0.6	2.02	0.84	8.34E+05	5.14E-05	3.3E-06	7.29E-03	3.2E-05	<LOD		137.2	1.2	2	3	3
GWS-0052	0.3	0.69	0.59	4.90E+05	6.56E-05	4.8E-06	7.29E-03	4.6E-05	<LOD		137.2	1.7	2	3	4
GWS-0002	2.9	6.37	2.73	2.73E+06	7.20E-05	1.6E-06	7.28E-03	1.8E-05	<LOD		137.3	0.7	1	3	3
GWS-0048	3.3	6.55	3.34	4.26E+06	6.05E-05	1.2E-06	7.28E-03	2.2E-05	<LOD		137.4	0.8	2	3	3
GWN-0002	8.4	12.62	6.61	8.95E+06	5.90E-05	7.2E-07	7.28E-03	3.3E-05	<LOD		137.4	1.3	3	3	4
GWS 0024	0.7	1.59	0.70	1.14E+06	7.18E-05	4.8E-06	7.28E-03	3.5E-05	<LOD		137.4	1.3	2	3	4
GWN-0015	0.9	1.39	0.53	1.32E+06	8.06E-05	2.9E-06	7.28E-03	3.7E-05	<LOD		137.5	1.4	3	3	3
GWN-0001	10.0	15.07	15.70	1.75E+07	7.79E-05	1.2E-06	7.27E-03	2.1E-05	<LOD		137.5	0.8	3	3	4
GWN-0022	5.8	8.04	4.17	4.26E+06	6.94E-05	1.1E-06	7.27E-03	3.0E-05	<LOD		137.5	1.1	3	3	2
GWN-0016	6.1	11.47	5.03	2.91E+06	6.21E-05	1.4E-06	7.27E-03	3.5E-05	<LOD		137.5	1.3	3	3	4
GWN-0004	2.2	7.64	3.01	3.05E+06	7.90E-05	1.3E-06	7.27E-03	3.7E-05	<LOD		137.5	1.4	3	3	2
GWN-0017	1.9	3.02	2.84	1.94E+06	7.06E-05	1.5E-06	7.27E-03	3.6E-05	<LOD		137.6	1.4	3	3	4
GWN-0032	2.4	8.03	2.37	3.16E+06	6.95E-05	1.2E-06	7.27E-03	3.0E-05	<LOD		137.6	1.1	3	3	1
GWS 0042	1.0	1.30	0.79	5.02E+06	6.30E-05	1.6E-06	7.27E-03	2.2E-05	<LOD		137.6	0.8	2	3	2
GWN-0077	0.5	0.93	0.55	5.52E+05	7.92E-05	8.5E-06	7.27E-03	4.8E-05	<LOD		137.6	1.8	3	3	4
GWN-0033	3.1	2.30	3.08	3.13E+06	8.27E-05	1.5E-06	7.27E-03	3.0E-05	<LOD		137.6	1.1	3	3	2
GWS-0047	3.7	10.08	4.69	4.71E+06	6.03E-05	1.1E-06	7.26E-03	2.1E-05	<LOD		137.7	0.8	2	3	4
GWN-0010	6.1	7.88	4.13	1.78E+06	7.01E-05	2.4E-06	7.26E-03	3.6E-05	<LOD		137.7	1.4	3	3	4

Table S3. U isotope measurements of samples

Sample Name	^{238}U *n* g/kg	24h U excretion	*n* g U/g creatinine	^{238}U intensity (cps)	^{234}U / ^{238}U	±	^{235}U / ^{238}U	±	^{236}U / ^{238}U	±	^{238}U / ^{235}U	± 2sd	InNI study[##]	Group φφ	DU Level [$$]
GWS-0056	1.3	3.44	1.38	2.46E+06	6.78E-05	1.9E-06	7.26E-03	2.5E-05	<LOD		137.7	0.9	2	3	4
GWN-0030	1.3	2.13	1.23	1.34E+06	7.98E-05	1.9E-06	7.26E-03	3.2E-05	<LOD		137.7	1.2	3	3	3
GWN-0058	1.2	3.23	1.54	6.05E+05	8.20E-05	2.6E-06	7.26E-03	3.6E-05	<LOD		137.7	1.4	3	3	4
GWN-0019	2.7	4.58	1.78	3.61E+06	7.70E-05	1.4E-06	7.25E-03	3.4E-05	<LOD		137.9	1.3	3	3	2
GWN-0091	4.2	11.58	6.99	3.30E+06	8.86E-05	3.4E-06	7.25E-03	3.0E-05	9.0E-06	1.1E-06	137.9	1.2	3	3	2
GWN-0040	1.1	1.76	0.91	1.02E+06	7.47E-05	3.0E-06	7.24E-03	3.7E-05	<LOD		138.0	1.4	3	3	3
GWN-0043	1.2	3.87	1.30	3.42E+06	7.08E-05	1.8E-06	7.24E-03	3.0E-05	<LOD		138.2	1.1	3	3	4
GWN-0083	3.4	10.13	4.42	5.74E+06	6.32E-05	2.3E-06	7.22E-03	3.8E-05	5.1E-06	6.3E-07	138.6	1.5	3	3	4
GWN-0068	0.5	0.83	0.35	5.47E+05	7.52E-05	4.2E-06	7.23E-03	5.8E-05	<LOD		138.4	2.2	3	3	2
GWS 0038	0.3	0.88	0.41	1.20E+06	8.31E-05	4.0E-06	7.20E-03	6.8E-05	<LOD		138.9	2.6	2	3	4
GWN-0075	0.4	0.60	0.34	5.11E+05	8.23E-05	5.3E-06	7.23E-03	7.1E-05	<LOD		138.4	2.7	3	3	4
GWS-0021	0.1	0.44	0.16	1.99E+05	1.22E-04	1.3E-05	7.16E-03	1.2E-04	<LOD		139.6	4.7	2	3	4
In-house urine reference samples (~1.4ppt)															
#2 +0.5ng IRM	4.1	0.50	0.05	4.54E+06	5.95E-05	8.8E-07	7.29E-03	1.2E-05	<LOD		137.2	0.4			
#3 +1ng IRMM	7.0	1.00	0.05	6.99E+06	5.91E-05	6.7E-07	7.28E-03	1.2E-05	1.5E-06	4.9E-07	137.4	0.4			
#4 +1.5ng IRM	12.9	1.50	0.05	1.41E+07	5.59E-05	4.0E-07	7.28E-03	1.0E-05	<LOD		137.4	0.4			
#5	0.9	0.00	0.05	9.93E+05	6.78E-05	2.4E-06	7.28E-03	3.5E-05	<LOD		137.3	1.3			
#6 +0.4ng IRM	3.3	0.40	0.05	2.50E+06	5.42E-05	1.4E-06	7.31E-03	2.6E-05	<LOD		136.8	1.0			
#7 +0.8ng IRM	5.5	0.80	0.05	9.36E+06	5.76E-05	8.2E-07	7.32E-03	3.4E-05	<LOD		136.5	1.3			
#8 +1.2ng IRM	8.1	1.20	0.05	7.81E+06	5.68E-05	1.0E-06	7.28E-03	3.3E-05	<LOD		137.4	1.2			
#9	1.4	0.00	0.05	2.52E+06	8.21E-05	2.0E-06	7.26E-03	3.0E-05	<LOD		137.8	1.2			
#10 +0.4ng IRI	4.1	0.40	0.05	7.10E+06	6.15E-05	8.5E-07	7.29E-03	2.8E-05	<LOD		137.2	1.1			
#11 +0.8ng IR	5.8	0.80	0.05	1.10E+07	5.64E-05	1.0E-06	7.26E-03	4.0E-05	<LOD		137.7	1.5			
#12 +1.2ng IR	9.8	1.20	0.05	1.59E+07	5.56E-05	7.3E-07	7.27E-03	2.7E-05	<LOD		137.5	1.0			
#13	0.9	0.00	0.05	1.53E+06	7.32E-05	3.5E-06	7.25E-03	4.6E-05	<LOD		138.0	1.7			
#14 +0.4ng IRI	4.6	0.40	0.05	3.12E+06	6.56E-05	2.6E-06	7.28E-03	3.7E-05	<LOD		137.3	1.4			
#16	1.9	0.00	0.05	2.38E+06	6.80E-05	2.3E-06	7.27E-03	3.1E-05	<LOD		137.6	1.2			

Note ##. 1, 2 twins; 2, 57 Seabees; 3, 97 from National Sample

Note φφ. -1, not deployed; 0, deployed controls without GWI; 1, GWI Syndrome 1; 2, GWI Syndrome 2; 3, GWI Syndrome 3

Note $$. 1, Level 1 direct exposure; 2, Level 2 indirect exposure; 3, Level 3 incidental exposure; 4, no exposure
