# Supplementary material for: Resolving whether inhalation of depleted uranium contributed to Gulf War Illness using high-sensitivity mass spectrometry
Source: Sci Rep. 2021 Feb 18;11:3218. doi: 10.1038/s41598-021-82535-3 (PMC7893152; doi:10.1038/s41598-021-82535-3)
Supplement: Supplementary file 3 — Supplementary Information 3. [file 41598_2021_82535_MOESM3_ESM.pdf]

Table S4. U isotope measurements of 2ppb 1%^{233}U-doped IRMM184 uranium solution

Sample Name	238U intensity (cps)	$^{234}U / ^{238}U$	±	$^{235}U / ^{238}U$	±	$^{236}U / ^{238}U$	±	^{236}U cps	$^{238}U / ^{235}U$	± 2 SE
IRMM184+1%233U	3.8E+07	5.419E-05	2.7E-07	7.258E-03	1.1E-05	1.3E-07	9.9E-08	3.8	137.78	0.40
IRMM184+1%233U	3.7E+07	5.468E-05	2.3E-07	7.259E-03	1.1E-05	1.3E-07	9.9E-08	3.7	137.76	0.40
IRMM184+1%233U	3.6E+07	5.522E-05	2.5E-07	7.259E-03	1.1E-05	1.3E-07	8.5E-08	3.1	137.75	0.40
IRMM184+1%233U	1.5E+07	5.545E-05	6.3E-07	7.273E-03	1.1E-05	3.4E-07	2.5E-07	3.6	137.49	0.43
IRMM184+1%233U	2.4E+07	5.458E-05	3.7E-07	7.269E-03	1.0E-05	2.0E-07	1.4E-07	3.3	137.58	0.40
IRMM184+1%233U	5.4E+07	5.410E-05	4.4E-07	7.262E-03	2.1E-05	8.6E-08	1.5E-07	7.9	137.71	0.78
IRMM184+1%233U	5.3E+07	5.433E-05	4.0E-07	7.260E-03	2.1E-05	8.9E-08	6.7E-08	3.5	137.74	0.78
IRMM184+1%233U	4.4E+07	5.498E-05	4.7E-07	7.257E-03	2.1E-05	1.1E-07	1.4E-07	6.0	137.80	0.79
IRMM184+1%233U	4.5E+07	5.428E-05	3.8E-07	7.265E-03	2.0E-05	1.0E-07	8.2E-08	3.7	137.64	0.76
IRMM184+1%233U	3.9E+07	5.448E-05	3.9E-07	7.268E-03	2.0E-05	1.2E-07	9.7E-08	3.8	137.59	0.77
IRMM184+1%233U	3.9E+07	5.385E-05	4.1E-07	7.261E-03	2.0E-05	1.2E-07	1.5E-07	5.9	137.72	0.76
IRMM184+1%233U	8.5E+07	5.252E-05	3.7E-07	7.252E-03	1.6E-05	5.3E-08	8.2E-08	7.0	137.89	0.60
IRMM184+1%233U	8.5E+07	5.276E-05	3.7E-07	7.260E-03	1.4E-05	5.3E-08	6.8E-08	5.8	137.73	0.54
IRMM184+1%233U	8.8E+07	5.298E-05	3.7E-07	7.256E-03	1.4E-05	5.1E-08	5.2E-08	4.6	137.83	0.52
IRMM184+1%233U	8.8E+07	5.304E-05	3.6E-07	7.255E-03	1.4E-05	5.1E-08	7.0E-08	6.2	137.83	0.53
IRMM184+1%233U	9.0E+07	5.201E-05	4.0E-07	7.257E-03	1.4E-05	5.0E-08	5.5E-08	4.9	137.79	0.51
IRMM184+1%233U	8.9E+07	5.230E-05	3.4E-07	7.257E-03	1.4E-05	5.0E-08	6.7E-08	5.9	137.80	0.53
IRMM184+1%233U	8.7E+07	5.276E-05	3.3E-07	7.265E-03	1.2E-05	5.1E-08	6.0E-08	5.2	137.65	0.46
IRMM184+1%233U	8.9E+07	5.227E-05	3.5E-07	7.270E-03	1.4E-05	5.0E-08	5.5E-08	4.9	137.55	0.52
IRMM184+1%233U	8.7E+07	5.316E-05	3.1E-07	7.265E-03	1.4E-05	5.1E-08	9.1E-08	7.9	137.64	0.51
IRMM184+1%233U	8.3E+07	5.281E-05	3.2E-07	7.265E-03	1.2E-05	5.4E-08	5.1E-08	4.2	137.64	0.47
IRMM184+1%233U	4.9E+07	5.207E-05	7.3E-07	7.271E-03	1.4E-05	9.6E-08	7.9E-08	3.9	137.54	0.52
IRMM184+1%233U	4.8E+07	5.290E-05	7.4E-07	7.257E-03	1.2E-05	9.9E-08	1.1E-07	5.1	137.79	0.44
IRMM184+1%233U	7.2E+07	5.403E-05	7.6E-07	7.259E-03	1.2E-05	6.3E-08	9.3E-08	6.7	137.77	0.44
IRMM184+1%233U	3.3E+07	5.449E-05	5.5E-07	7.266E-03	3.0E-05	1.5E-07	9.1E-08	3.0	137.62	1.15
IRMM184+1%233U	2.8E+07	5.417E-05	5.7E-07	7.262E-03	3.0E-05	1.7E-07	1.4E-07	3.8	137.70	1.14
IRMM184+1%233U	2.8E+07	5.317E-05	6.4E-07	7.255E-03	3.0E-05	1.7E-07	1.1E-07	3.2	137.83	1.16
IRMM184+1%233U	3.3E+07	5.358E-05	5.4E-07	7.258E-03	3.0E-05	1.5E-07	1.1E-07	3.5	137.78	1.14
IRMM184+1%233U	3.2E+07	5.391E-05	4.8E-07	7.263E-03	3.0E-05	1.5E-07	1.5E-07	4.9	137.68	1.13
IRMM184+1%233U	2.8E+07	5.408E-05	5.0E-07	7.257E-03	3.0E-05	1.7E-07	1.1E-07	3.1	137.80	1.13
IRMM184+1%233U	1.9E+07	5.438E-05	7.9E-07	7.270E-03	3.0E-05	2.6E-07	1.7E-07	3.3	137.56	1.13
IRMM184+1%233U	1.9E+07	5.411E-05	5.2E-07	7.263E-03	3.0E-05	2.6E-07	1.9E-07	3.6	137.68	1.14
IRMM184+1%233U	2.0E+07	5.347E-05	6.0E-07	7.262E-03	3.1E-05	2.5E-07	2.0E-07	3.9	137.71	1.18
IRMM184+1%233U	2.1E+07	5.410E-05	5.7E-07	7.258E-03	3.0E-05	2.3E-07	2.0E-07	4.1	137.77	1.14
IRMM184+1%233U	2.0E+07	5.345E-05	6.4E-07	7.269E-03	3.1E-05	2.4E-07	2.0E-07	4.0	137.57	1.15
IRMM184+1%233U	3.0E+07	5.208E-05	6.6E-07	7.263E-03	3.3E-05	1.6E-07	1.4E-07	4.1	137.68	1.25
IRMM184+1%233U	3.0E+07	5.362E-05	5.4E-07	7.230E-03	3.3E-05	1.6E-07	2.0E-07	6.0	138.30	1.25
IRMM184+1%233U	3.0E+07	5.426E-05	5.8E-07	7.245E-03	3.3E-05	1.6E-07	1.9E-07	5.7	138.02	1.25
IRMM184+1%233U	3.4E+07	5.318E-05	5.8E-07	7.275E-03	3.3E-05	1.4E-07	1.2E-07	4.1	137.45	1.26
IRMM184+1%233U	1.1E+07	5.255E-05	7.0E-07	7.301E-03	3.4E-05	4.4E-07	2.9E-07	3.3	136.97	1.27
IRMM184+1%233U	4.1E+07	5.408E-05	5.6E-07	7.247E-03	3.3E-05	1.2E-07	7.5E-08	3.1	137.99	1.26
IRMM184+1%233U	4.0E+07	5.329E-05	6.2E-07	7.256E-03	3.3E-05	1.2E-07	1.1E-07	4.4	137.82	1.25
IRMM184+1%233U	3.0E+07	5.443E-05	5.6E-07	7.262E-03	3.3E-05	1.6E-07	1.2E-07	3.7	137.70	1.25
IRMM184+1%233U	2.9E+07	5.371E-05	5.8E-07	7.273E-03	3.3E-05	1.7E-07	1.5E-07	4.4	137.49	1.25
IRMM184+1%233U	3.0E+07	5.349E-05	5.7E-07	7.269E-03	3.3E-05	1.6E-07	1.1E-07	3.2	137.58	1.24
IRMM184+1%233U	3.0E+07	5.247E-05	5.6E-07	7.266E-03	3.0E-05	1.6E-07	1.2E-07	3.5	137.63	1.12
IRMM184+1%233U	3.3E+07	5.273E-05	4.8E-07	7.277E-03	2.8E-05	1.5E-07	1.2E-07	4.0	137.41	1.07
IRMM184+1%233U	4.6E+07	5.405E-05	5.2E-07	7.258E-03	2.8E-05	1.0E-07	6.5E-08	3.0	137.79	1.07
IRMM184+1%233U	4.5E+07	5.409E-05	5.7E-07	7.251E-03	2.8E-05	1.0E-07	8.2E-08	3.7	137.90	1.07
IRMM184+1%233U	4.5E+07	5.309E-05	5.9E-07	7.257E-03	2.8E-05	1.1E-07	1.0E-07	4.7	137.79	1.06
IRMM184+1%233U	3.3E+07	5.372E-05	5.7E-07	7.283E-03	2.9E-05	1.5E-07	1.3E-07	4.3	137.30	1.09
IRMM184+1%233U	3.2E+07	5.300E-05	5.5E-07	7.279E-03	2.8E-05	1.5E-07	9.7E-08	3.1	137.39	1.07
IRMM184+1%233U	3.4E+07	5.417E-05	6.5E-07	7.268E-03	2.8E-05	1.4E-07	1.1E-07	3.7	137.59	1.07
IRMM184+1%233U	3.3E+07	5.379E-05	5.5E-07	7.272E-03	2.8E-05	1.5E-07	1.1E-07	3.7	137.52	1.07
IRMM184+1%233U	5.5E+07	5.490E-05	6.1E-07	7.243E-03	2.9E-05	8.6E-08	7.9E-08	4.3	138.07	1.10
IRMM184+1%233U	5.8E+07	5.480E-05	5.0E-07	7.227E-03	2.8E-05	8.1E-08	8.7E-08	5.0	138.38	1.07
IRMM184+1%233U	4.5E+07	5.430E-05	5.2E-07	7.266E-03	2.8E-05	1.1E-07	9.0E-08	4.0	137.63	1.06
IRMM184+1%233U	5.7E+07	5.338E-05	4.6E-07	7.289E-03	3.3E-05	8.2E-08	9.9E-08	5.7	137.20	1.23

Sample Name	238U intensity (cps)	$^{234}U / ^{238}U$	±	$^{235}U / ^{238}U$	±	$^{236}U / ^{238}U$	±	^{236}U cps	$^{238}U / ^{235}U$	± 2 SE
IRMM184+1%233U	5.6E+07	5.421E-05	5.5E-07	7.275E-03	2.6E-05	8.3E-08	1.3E-07	7.2	137.46	0.98
IRMM184+1%233U	5.6E+07	5.335E-05	4.2E-07	7.274E-03	2.6E-05	8.4E-08	5.1E-08	2.8	137.48	0.97
IRMM184+1%233U	5.2E+07	5.378E-05	4.4E-07	7.263E-03	2.6E-05	9.1E-08	7.5E-08	3.9	137.69	0.97
IRMM184+1%233U	5.0E+07	5.387E-05	4.4E-07	7.274E-03	2.6E-05	9.4E-08	8.9E-08	4.4	137.48	0.98
IRMM184+1%233U	5.1E+07	5.400E-05	5.4E-07	7.272E-03	2.7E-05	9.1E-08	6.4E-08	3.3	137.51	1.01
IRMM184+1%233U	5.4E+07	5.430E-05	4.7E-07	7.250E-03	2.7E-05	8.6E-08	7.7E-08	4.2	137.93	1.02
IRMM184+1%233U	5.8E+07	5.420E-05	5.1E-07	7.233E-03	2.7E-05	8.1E-08	6.9E-08	4.0	138.25	1.02
IRMM184+1%233U	5.3E+07	5.475E-05	4.9E-07	7.255E-03	2.6E-05	8.9E-08	9.6E-08	5.0	137.84	0.97
IRMM184+1%233U	5.4E+07	5.426E-05	4.6E-07	7.247E-03	2.6E-05	8.6E-08	8.3E-08	4.5	137.98	0.99
IRMM184+1%233U	5.4E+07	5.492E-05	5.2E-07	7.233E-03	3.3E-05	8.7E-08	7.7E-08	4.1	138.25	1.26
IRMM184+1%233U	4.8E+07	5.346E-05	4.5E-07	7.254E-03	2.6E-05	9.7E-08	8.5E-08	4.1	137.85	0.99
IRMM184+1%233U	4.7E+07	5.442E-05	5.1E-07	7.263E-03	2.6E-05	1.0E-07	7.3E-08	3.5	137.68	0.98
IRMM184+1%233U	4.8E+07	5.405E-05	5.1E-07	7.288E-03	4.2E-05	9.8E-08	8.6E-08	4.1	137.20	1.59
IRMM184+1%233U	4.9E+07	5.170E-05	1.7E-06	7.287E-03	3.6E-05	9.6E-08	8.1E-08	4.0	137.24	1.35
IRMM184+1%233U	4.8E+07	5.199E-05	1.7E-06	7.279E-03	3.5E-05	9.8E-08	5.8E-08	2.8	137.38	1.33
IRMM184+1%233U	4.0E+07	5.237E-05	1.7E-06	7.262E-03	3.6E-05	1.2E-07	1.1E-07	4.5	137.70	1.35
IRMM184+1%233U	4.1E+07	5.202E-05	1.7E-06	7.273E-03	3.5E-05	1.2E-07	1.1E-07	4.6	137.49	1.33
IRMM184+1%233U	4.3E+07	5.235E-05	1.7E-06	7.281E-03	3.7E-05	1.1E-07	1.1E-07	4.7	137.34	1.39
IRMM184+1%233U	4.0E+07	5.244E-05	1.7E-06	7.285E-03	3.6E-05	1.2E-07	9.1E-08	3.7	137.27	1.34
IRMM184+1%233U	4.0E+07	5.241E-05	1.7E-06	7.267E-03	3.6E-05	1.2E-07	9.3E-08	3.8	137.61	1.37
IRMM184+1%233U	4.1E+07	5.251E-05	1.7E-06	7.269E-03	3.5E-05	1.2E-07	8.3E-08	3.4	137.57	1.33
IRMM184+1%233U	6.0E+07	5.538E-05	1.8E-06	7.235E-03	3.5E-05	7.7E-08	8.6E-08	5.2	138.21	1.33
IRMM184+1%233U	6.0E+07	5.449E-05	1.8E-06	7.233E-03	3.5E-05	7.7E-08	6.9E-08	4.1	138.25	1.34
IRMM184+1%233U	5.3E+07	5.527E-05	1.8E-06	7.238E-03	3.5E-05	8.9E-08	7.9E-08	4.2	138.16	1.33
IRMM184+1%233U	5.4E+07	5.592E-05	1.8E-06	7.236E-03	3.5E-05	8.6E-08	1.0E-07	5.6	138.19	1.34
IRMM184+1%233U	6.4E+07	5.197E-05	6.8E-07	7.259E-03	1.7E-05	7.2E-08	7.2E-08	4.6	137.76	0.63
IRMM184+1%233U	6.5E+07	5.250E-05	6.4E-07	7.272E-03	1.7E-05	7.0E-08	7.6E-08	5.0	137.52	0.65
IRMM184+1%233U	6.6E+07	5.249E-05	6.8E-07	7.260E-03	1.7E-05	7.0E-08	6.8E-08	4.5	137.73	0.66
IRMM184+1%233U	5.8E+07	5.212E-05	7.2E-07	7.259E-03	1.7E-05	8.0E-08	7.8E-08	4.5	137.75	0.65
IRMM184+1%233U	5.2E+07	5.239E-05	6.7E-07	7.265E-03	1.6E-05	9.1E-08	1.1E-07	5.8	137.64	0.61
IRMM184+1%233U	4.7E+07	5.376E-05	6.8E-07	7.263E-03	2.7E-05	1.0E-07	8.4E-08	4.0	137.69	1.03
IRMM184+1%233U	4.7E+07	5.335E-05	7.0E-07	7.257E-03	1.6E-05	1.0E-07	1.1E-07	5.1	137.80	0.63
IRMM184+1%233U	5.3E+07	5.254E-05	3.4E-07	7.273E-03	2.6E-05	8.8E-08	8.9E-08	4.7	137.49	0.97
IRMM184+1%233U	5.3E+07	5.204E-05	3.6E-07	7.261E-03	2.7E-05	8.8E-08	1.3E-07	6.7	137.73	1.01
IRMM184+1%233U	4.9E+07	5.238E-05	4.5E-07	7.257E-03	2.6E-05	9.7E-08	7.6E-08	3.7	137.80	0.99
IRMM184+1%233U	5.0E+07	5.309E-05	4.2E-07	7.255E-03	2.6E-05	9.4E-08	7.9E-08	4.0	137.84	0.97
IRMM184+1%233U	4.1E+07	5.254E-05	4.0E-07	7.264E-03	2.6E-05	1.2E-07	1.1E-07	4.4	137.67	0.97
IRMM184+1%233U	4.0E+07	5.193E-05	5.3E-07	7.264E-03	2.6E-05	1.2E-07	8.6E-08	3.5	137.67	0.97
IRMM184+1%233U	1.2E+07	5.186E-05	5.7E-07	7.235E-03	1.1E-05	4.0E-07	2.6E-07	3.2	138.21	0.42
IRMM184+1%233U	1.2E+07	5.316E-05	5.2E-07	7.254E-03	1.5E-05	4.0E-07	2.6E-07	3.2	137.86	0.57
IRMM184+1%233U	1.2E+07	5.338E-05	4.3E-07	7.261E-03	1.2E-05	4.1E-07	2.8E-07	3.4	137.72	0.45
IRMM184+1%233U	5.4E+07	5.216E-05	1.7E-07	7.256E-03	9.7E-06	8.6E-08	1.2E-07	6.5	137.83	0.37
IRMM184+1%233U	5.5E+07	5.226E-05	2.7E-07	7.262E-03	9.1E-06	8.4E-08	6.6E-08	3.7	137.70	0.35
IRMM184+1%233U	5.6E+07	5.243E-05	3.5E-07	7.255E-03	8.5E-06	8.4E-08	1.2E-07	6.4	137.84	0.32
IRMM184+1%233U	5.3E+07	5.251E-05	4.3E-07	7.271E-03	1.8E-05	8.9E-08	1.2E-07	6.3	137.53	0.67
IRMM184+1%233U	5.2E+07	5.175E-05	2.3E-07	7.263E-03	1.1E-05	9.0E-08	7.8E-08	4.0	137.68	0.43
IRMM184+1%233U	5.3E+07	5.274E-05	2.7E-07	7.256E-03	9.5E-06	8.9E-08	1.1E-07	5.7	137.81	0.36
IRMM184+1%233U	5.3E+07	5.285E-05	3.7E-07	7.272E-03	9.8E-06	8.9E-08	9.3E-08	4.9	137.52	0.37
IRMM184+1%233U	5.4E+07	5.270E-05	3.2E-07	7.261E-03	8.9E-06	8.6E-08	1.1E-07	6.0	137.72	0.34
IRMM184+1%233U	5.4E+07	5.320E-05	2.5E-07	7.264E-03	8.5E-06	8.7E-08	7.6E-08	4.1	137.67	0.32
IRMM184+1%233U	3.0E+07	5.601E-05	4.0E-07	7.221E-03	4.0E-05	1.6E-07	1.1E-07	3.1	138.49	1.52
IRMM184+1%233U	2.8E+07	5.595E-05	4.8E-07	7.255E-03	4.0E-05	1.7E-07	1.5E-07	4.1	137.84	1.52
IRMM184+1%233U	2.9E+07	5.633E-05	6.7E-07	7.251E-03	3.9E-05	1.6E-07	1.6E-07	4.8	137.92	1.50
IRMM184+1%233U	3.9E+07	5.315E-05	2.9E-07	7.236E-03	3.9E-05	1.2E-07	9.6E-08	3.7	138.20	1.51
IRMM184+1%233U	3.3E+07	5.320E-05	5.6E-07	7.271E-03	4.0E-05	1.4E-07	8.8E-08	3.0	137.53	1.50
IRMM184+1%233U	3.2E+07	5.294E-05	3.5E-07	7.249E-03	3.9E-05	1.5E-07	1.5E-07	4.7	137.94	1.49
IRMM184+1%233U	2.6E+07	5.440E-05	3.5E-07	7.270E-03	4.1E-05	1.9E-07	1.4E-07	3.6	137.55	1.56
IRMM184+1%233U	2.5E+07	5.418E-05	4.3E-07	7.274E-03	4.1E-05	1.9E-07	1.5E-07	3.7	137.47	1.54
IRMM184+1%233U	2.2E+07	5.443E-05	4.8E-07	7.272E-03	4.0E-05	2.2E-07	2.4E-07	5.2	137.51	1.50
IRMM184+1%233U	5.8E+07	5.217E-05	5.1E-07	7.263E-03	1.6E-05	8.0E-08	5.2E-08	3.1	137.68	0.60

Sample Name	238U intensity (cps)	^{234}U / ^{238}U	±	^{235}U / ^{238}U	±	^{236}U / ^{238}U	±	^{236}U cps	^{238}U / ^{235}U	± 2 SE
IRMM184+1%233U	5.9E+07	5.227E-05	3.9E-07	7.261E-03	1.4E-05	7.9E-08	5.9E-08	3.5	137.71	0.53
IRMM184+1%233U	6.0E+07	5.507E-05	2.3E-06	7.297E-03	3.8E-05	7.7E-08	7.9E-08	4.7	137.03	1.44
IRMM184+1%233U	6.1E+07	5.520E-05	2.3E-06	7.299E-03	3.7E-05	7.6E-08	5.1E-08	3.1	137.01	1.38
IRMM184+1%233U	5.8E+07	5.408E-05	2.2E-06	7.253E-03	4.1E-05	8.0E-08	5.3E-08	3.1	137.88	1.58
IRMM184+1%233U	5.6E+07	5.467E-05	2.2E-06	7.242E-03	3.9E-05	8.3E-08	6.3E-08	3.5	138.09	1.50
IRMM184+1%233U	5.7E+07	5.054E-05	2.1E-06	7.224E-03	4.3E-05	8.1E-08	7.5E-08	4.3	138.44	1.65
IRMM184+1%233U	5.6E+07	5.120E-05	2.1E-06	7.263E-03	3.8E-05	8.4E-08	6.8E-08	3.8	137.69	1.43
IRMM184+1%233U	5.7E+07	5.048E-05	2.1E-06	7.258E-03	3.7E-05	8.1E-08	6.7E-08	3.8	137.78	1.40
Notes: all uncertainties are 1σ unless otherwise noted; variations in intensity reflect solution uptake and instrument sensitivity variations										
